# Discovery of *N*‑Acylhydrazone Derivatives as ROCK Inhibitors: A Journey from Virtual Screening and Structure-Based De Novo Design to the Identification of ROCK2 Selective Inhibitors and Beyond

**DOI:** 10.1021/acsbiomedchemau.5c00246

**Published:** 2026-01-27

**Authors:** Pedro de Sena Murteira Pinheiro, Lucas Silva Franco, Raysa Magali Pillpe-Meza, Bárbara da Silva Mascarenhas de Jesus, Gabrielli Ayumi Ito Martins, Wesley Leandro Gouveia, Daniel Alencar Rodrigues, Marina Amaral Alves, Lídia Moreira Lima

**Affiliations:** † Laboratório de Avaliação e Síntese de Substâncias Bioativas (LASSBio), Instituto de Ciências Biomédicas, 215033 Universidade Federal do Rio de Janeiro, Rio de Janeiro, Rio de Janeiro 21941-902, Brazil; ‡ Programa de Pós-Graduação em Farmacologia e Química Medicinal, Instituto de Ciências Biomédicas, Universidade Federal do Rio de Janeiro, Rio de Janeiro, Rio de Janeiro 21941-902, Brazil; § School of Pharmacy and Biomolecular Sciences (PBS), 8863 Royal College of Surgeons in Ireland, 1st Floor Ardilaun House Block B 111 St Stephen’s Green, Dublin 2 D02 YN77, Ireland; ∥ Walter Mors Institute of Research on Natural Products, 28125 Universidade Federal do Rio de Janeiro, Rio de Janeiro, Rio de Janeiro 21941-902, Brazil

**Keywords:** Rho kinase, metastasis, AGC family, kinase inhibitors, computer-aided drug design

## Abstract

Rho-associated coiled-coil containing kinases (ROCK1 and ROCK2) are central regulators of actin cytoskeleton organization and cell contractility under physiological conditions. Dysregulation of ROCK signaling contributes to aberrant cell migration, invasion, and tissue remodeling, positioning these kinases as attractive therapeutic targets in cancer and fibrotic diseases. In this work, we report the discovery of *N*-acylhydrazone (NAH) derivatives as potent and selective ROCK inhibitors, integrating structure-based virtual screening (SBVS), de novo design, and biological evaluation. Initial hit identification from the LASSBio Chemical Library revealed three inhibitors (LASSBio-1828 (**1**), LASSBio-1829 (**2**), and LASSBio-1919 (**3**)), which guided the rational design of a virtual library of 321 NAH analogues. Docking-based prioritization, synthesis, and SAR exploration yielded compounds with low nanomolar potency, among which LASSBio-2360 (**12**), LASSBio-2380 (**17**), and LASSBio-2382 (**18**) exhibited dual ROCK1/2 inhibition (IC_50_ values in the 1–15 nM range), while LASSBio-2389 (**21**) showed remarkable ROCK2 selectivity (IC_50_ = 0.051 μM; 21-fold vs ROCK1 - IC_50_ = 1.143 μM) and minimal inhibition of other related kinases at 500 nM. Molecular dynamics simulations demonstrated that **21** stabilizes the DFG-out conformation of ROCK2, providing a structural rationale for isoform selectivity. In vitro studies using MDA-MB-231 triple-negative breast cancer cells confirmed that compounds **12**, **17**, **18**, and **21** inhibit migration more effectively than fasudil and comparably to belumosudil. Altogether, this work identifies NAH as a privileged scaffold for ROCK inhibition, delineates the molecular determinants of ROCK2 selectivity, and highlights new chemical leads for the development of antimetastatic and antifibrotic agents targeting the Rho/ROCK pathway.

## Graphical abstract

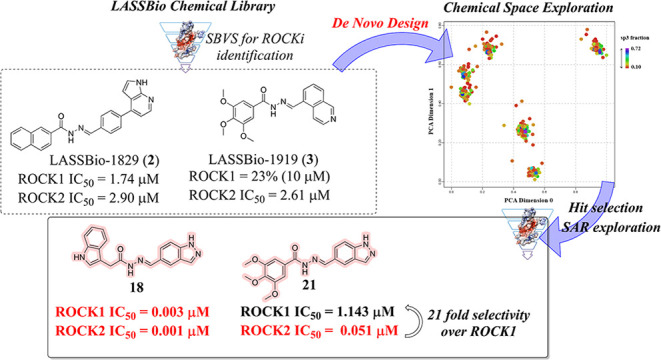

## Introduction

Rho-associated coiled-coil containing kinases (ROCKs) are serine/threonine kinases that act as principal effectors of the small GTPase RhoA and regulate actin–myosin contractility, cell shape, motility, adhesion, and cytokinesis. ROCK signaling controls cytoskeletal dynamics through phosphorylation of substrates such as myosin light-chain phosphatase (MYPT1) and LIM kinases, thereby modulating cell contractility, migration, and extracellular matrix interactions.^1,2^ Owing to their central role in these processes, ROCK kinases have emerged as therapeutically relevant targets, being implicated in cancer progression, fibrosis, cardiovascular, and neurological disorders.^3–7^

Structurally, ROCK1 and ROCK2 share a highly conserved *N*-terminal kinase domain, a central coiled-coil region that mediates dimerization and contains the Rho-binding domain, and a *C*-terminal pleckstrin homology domain that contributes to autoregulation and membrane association.^6,8,9^ High-resolution crystallographic data for ROCK kinases show that most small-molecule inhibitors bind in the ATP site of the kinase domain. Available structures indicate that the kinases adopt a canonical active state (DFG-in/α*C*-in) and, in general, small-molecule inhibitors act as type 1 ATP-competitive inhibitors.^10–13^

Small-molecule inhibitors of Rho-associated kinases (ROCKs) encompass diverse chemical classes and have been explored across multiple therapeutic areas, reflecting the broad physiological and pathological significance of ROCK signaling. Fasudil, a nonselective ROCK1/2 inhibitor, represents a historical milestone as the first kinase inhibitor ever approved for clinical use, preceding even the introduction of tyrosine kinase inhibitors such as imatinib.^14^ It was approved in Japan (1995) and China (2006) for the treatment of cerebral vasospasm,^15,16^ and was also investigated in other conditions, such as amyotrophic lateral sclerosis,^17^ Parkinson’s disease,^18^ cancer and beyond.^3^ In ophthalmology, ripasudil and netarsudil are approved for the treatment of glaucoma and ocular hypertension.^19^ More recently, the selective ROCK2 inhibitor belumosudil received FDA approval in 2021 for chronic graft-versus-host disease, marking the first isoform-selective ROCK inhibitor to reach the clinic.^20^ Furthermore, next-generation inhibitors such as verosudil, sovesudil and zelasudil are currently in clinical trials for cancer, fibrotic, cardiovascular, neurological and inflammatory diseases, underscoring the expanding therapeutic scope of ROCK inhibition.^3,4^ The chemical structure of the mentioned ROCK inhibitors are shown in Figure 1.

**Figure 1:**
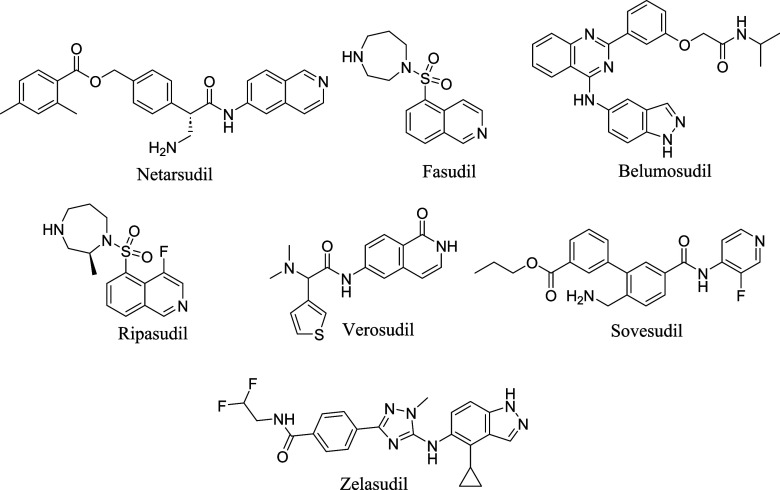
Chemical structure of ROCK inhibitors.

There is growing preclinical and translational evidence linking ROCK activity to tumor biology, ROCK signaling influences cancer-relevant processes including cell invasion, migration, angiogenesis, mechanotransduction, and therapy resistance. Experimental inhibition of ROCK reduces metastatic behaviors and impairs tumor progression, supporting ROCK as a candidate therapeutic target in oncology.^4,21,22^ Multiple ROCK inhibitors are being evaluated preclinically and in early clinical studies for oncologic indications or are being repurposed from vascular/ophthalmic uses toward cancer research.^3,21^

Taken together, ROCK kinases combine a well-defined enzymatic domain amenable to small-molecule inhibition with pleiotropic control over cytoskeletal and microenvironmental mechanisms that drive malignant progression. These features make ROCK a tractable and biologically rational target for antimetastatic and combination cancer therapies, although isoform selectivity, context-dependent effects and potential on-target toxicities remain key challenges for clinical translation.

In this work, we envisioned the usage of our internal collection of small-molecule drug candidates, i.e., the LASSBio Chemical Library,^23^ in an initial hit identification campaign through SBVS. Next, based on the identified hits, we applied a de novo design strategy using the NAH framework as a central core linking the hinger binding motif with the terminal aromatic rings that occupy the ROCK affinity pocket.^12^ Our approach led to the identification of potent ROCK1/2 dual inhibitors (**12**, **17** and **18**), as well as ROCK2-selective inhibitors (**21**). To our knowledge, this is the first study reporting NAH derivatives as ROCK inhibitors.

## Results and Discussion

### Virtual Screening Campaign against ROCK

Through the recently reported pharmacophoric map for the identification of ROCK inhibitors in SBVS campaigns,^12^ we applied the same methodology using our in-house chemical library, the LASSBio Chemical Library.^23^ In this way, we were able to identify 3 hits: LASSBio-1828 (**1**),^24^ LASSBio-1829 (**2**),^25^ and LASSBio-1919 (**3**).^26^ LASSBio-1828 (**1**) showed 43% inhibition of ROCK1 at 10 μM with an estimated IC_50_ of 44 μM, while it showed a slightly less significant inhibition of ROCK2 (27%) at the same concentration. In contrast, LASSBio-1829 (**2**), which is structurally similar to LASSBio-1828 (**1**), differing only at the hinge binder motif, showed IC_50_ values with a 1-digit micromolar range for both ROCK isoforms (ROCK1 IC_50_ = 1.74 μM; ROCK2 IC_50_ = 2.90 μM). LASSBio-1919 (**3**), on the other hand, showed selectivity for ROCK2 inhibition with an IC_50_ value of 2.61 μM while showing 23% max inhibition of ROCK1 at 10 μM. In Figure 2D, the interaction mode of LASSBio-1829 (**2**) in ROCK1 is shown, in which the 7-azaindole moiety forms a bidentate hydrogen bond interaction with the main chain of Met156 and Glu154, located at the hinge region. It is important to note that, compared to the cocrystallized ligand in the PDB 6E9W, this ligand can access the affinity pocket, forming a cation-π interaction with the catalytic Lys105 residue. In contrast to compounds **1** and **2**, **3** features a shorter linker and an isoquinoline hinge-binding motif. Docking and subsequent pose inspection indicate that **3** can still be accommodated in the ATP-binding site, with the isoquinoline ring positioned at the hinge region to maintain a canonical hinge-anchoring interaction pattern (Figure 2E). Notably, the predicted interaction pattern and overall pose of **3** were essentially analogous to that observed for compound **21**, which will be discussed further. The identified hits from LASSBio Chemical Library do not present conformational freedom to access this cavity. From our SBVS some major points were depicted: (i) focusing on hinge binders that can perform bidentate hydrogen bonding interactions, as the 7-azaindole moiety, seems to be more promising; (ii) the *N*-acylhydrazone moiety is an interesting linker, but it needs to be more flexible; (iii) the trimethoxyphenyl moiety resulted in ROCK2 selectivity.

**Figure 2:**
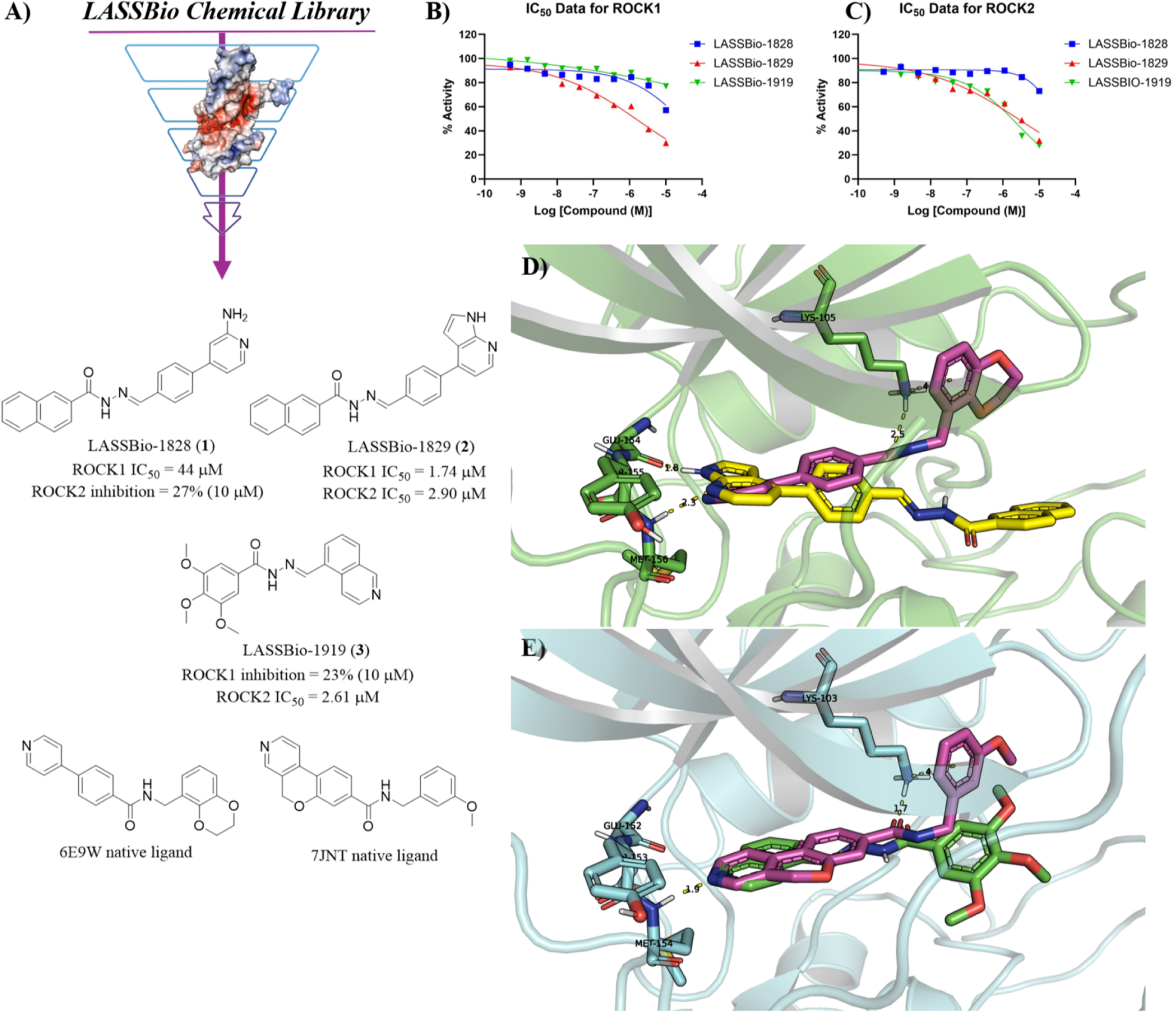
Identification of *N*-acylhydrazone derivatives as ROCK inhibitors. (A) SBVS from LASSBio Chemical Library leading to identification of LASSBio-1828 (**1**), LASSBio-1829 (**2**) and LASSBio-1919 (**3**) as ROCK inhibitors. (B) IC_50_ curves for ROCK1 inhibition. (C) IC_50_ curves for ROCK2 inhibition. (D) Calculated interaction mode of LASSBio-1829 (**2**) (carbons in yellow) in ROCK1 superposed with the native ligand (carbons in magenta) of the 6E9W crystal structure (resolution: 2.96 Å). (E) Calculated interaction mode of LASSBio-1919 (**3**) (carbons in green) in ROCK2 superposed with the native ligand (carbons in magenta) of the 7JNT crystal structure (resolution: 2.21 Å).

### De Novo Design of New *N*-Acylhydrazone Derivatives

Based on the three identified hits and the information gathered in the virtual screening campaign, we conducted a de novo design strategy. This strategy was focused on the NAH framework as a linker, bridging the hinge binding core with the terminal aromatic system. Thus, allowing the effective hydrogen bonding interactions with the hinge, while performing a cation-π interaction with the catalytic lysine in the affinity pocket of ROCK1/2.

The de novo design approach was focused on our internal collection of reagents and fragments. We built a KNIME^27^ workflow to filter and collect carboxylic acids. These were transformed into the corresponding hydrazides using the RDKit “Two Component Reaction” node through a SMARTS pattern for the reaction (Figure 3A). Next, aldehydes of interest (isoquinoline-5-carbaldehyde (**6**), quinoline-4-carbaldehyde (**7**), 4-(pyridin-4-yl)­benzaldehyde (**8**), isonicotinaldehyde (**9**), 1H-pyrrolo­[2,3-*b*]­pyridine-5-carbaldehyde (**10**), 1H-pyrrolo­[2,3-*b*]­pyridine-3-carbaldehyde (**11**)) were selected from our catalog and condensed with the generated hydrazides in a second reaction using also the RDKit “Two Component Reaction” node through a SMARTS pattern for the reaction (Figure 3A). These resulted in 1282 unique virtual NAH derivatives that were subsequently filtered based on sp^3^ fraction (≥0.1) and number of stereo centers (= 0), resulting in 321 derivatives (Figure 3A), which were analyzed by means of chemical space (Figure 3B) and exported in SDF format for three-dimensional geometry optimization in Spartan’24, followed by molecular docking studies using GOLD’2025.1.0.

**Figure 3:**
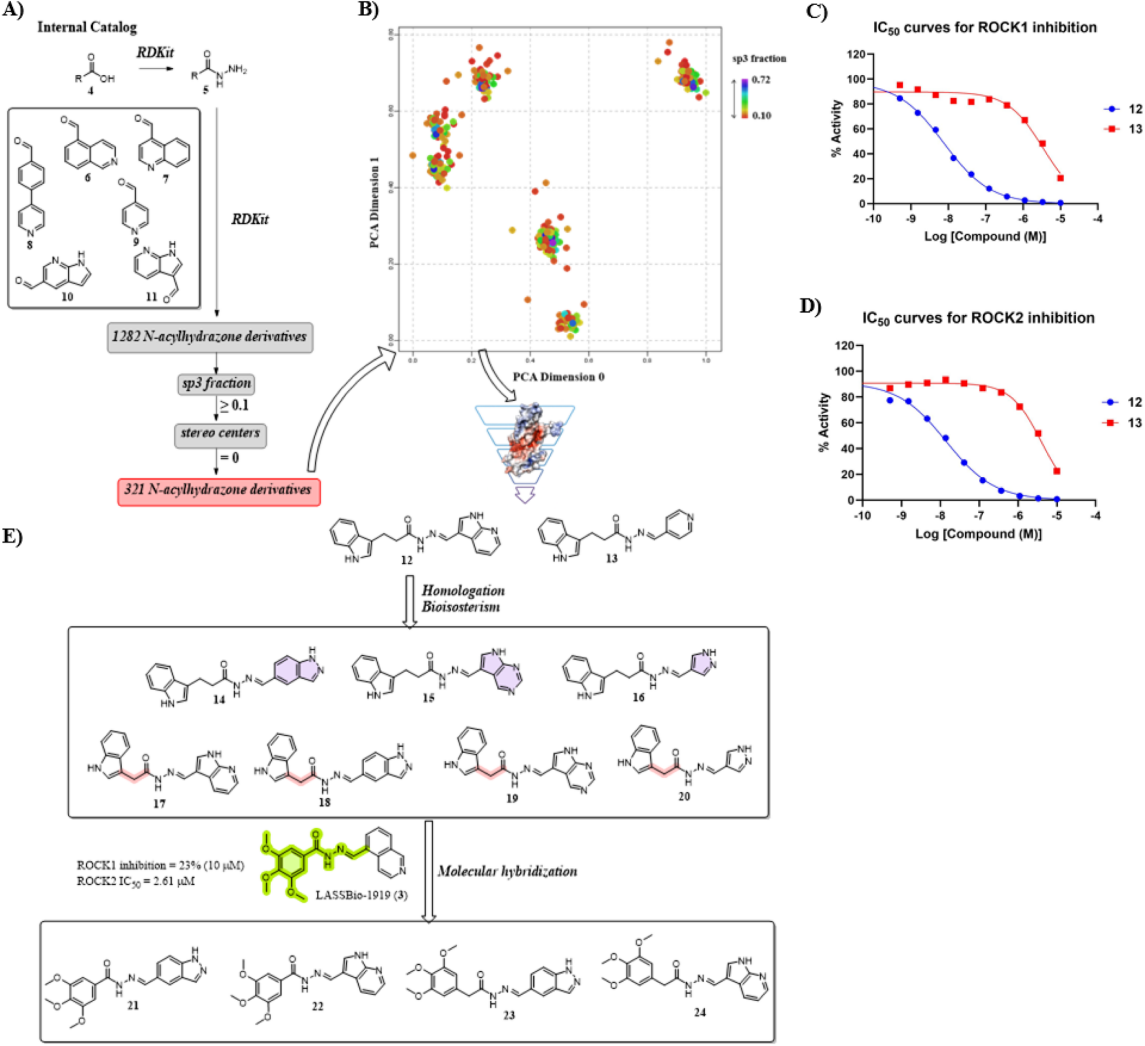
De novo design approach of *N*-acylhydrazone derivatives for ROCK inhibition. (A) Workflow representation using RDKit to generate the virtual library of *N*-acylhydrazone derivatives. (B) Chemical space analysis of the resulting 321 *N*-acylhydrazone derivatives. (C) IC_50_ curves for ROCK1 inhibition of compounds **12** and **13**. (D) IC_50_ curves for ROCK1 inhibition of compounds **12** and **13**. (E) Chemical structures of **12** and **13** and design strategy for SAR exploration (**14**-**24**).

In this de novo design approach, we intentionally explored both phenyl-hydrazone linkers and “direct” hydrazone linkers (i.e., hinge binders connected directly to the NAH unit without an intervening phenyl ring), motivated by the initial hit LASSBio-1919 (**3**), which features an isoquinoline hinge binder directly attached to the NAH framework (Figure 3A). Importantly, during docking-based prioritization of the virtual de novo library, the candidates that most consistently satisfied the key pharmacophoric requirements in the ROCK ATP site—productive hinge anchoring together with the expected interaction toward the affinity pocket—were predominantly those lacking the phenyl linker.

Keeping in mind our goal, i.e., identifying a small molecule capable of binding simultaneously at the hinge region and the affinity pocket, we selected two promising NAH derivatives from our de novo design approach. According to our docking protocol, **12** and **13** are both capable of performing the key interactions with ROCK1 and ROCK2, as a consequence of their higher flexibility, a necessary feature that was already anticipated in the virtual screening campaign. As an example, the interaction mode of **12** in ROCK1 and ROCK2 is shown in Figure 4A,B, in which the only difference between **12** and **13** regards the binding mode at the hinge. While **13** forms a monodentate hydrogen bond through the pyridine ring, **12** forms a bidentate hydrogen bond through the 7-azaindole core. Next, **12** and **13** were synthesized (Scheme 1, see the next section for more details) and evaluated against ROCK1/2 (Figure 3C,D, Table 1) in order to validate our approach. Compound **13** exhibited IC_50_ values of 3.39 μM and 3.98 μM against ROCK1 and ROCK2, respectively, whereas **12** was 450- and 280-fold more potent, with IC_50_ values of 7.5 nM and 14.1 nM for ROCK1 and ROCK2, respectively. Ultimately, this not only validates our approach but also clearly indicates that the design of new derivatives should prioritize the bidentate hydrogen bonding in the hinge while maintaining some level of sp3 fraction at the NAH framework.

**Scheme 1:**
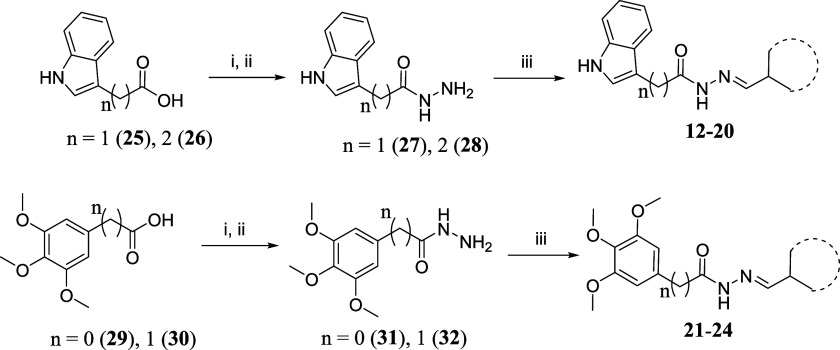
Reaction Conditions: (i) MeOH, H_2_SO_4_, Reflux, Overnight; (ii) EtOH, NH_2_NH_2_.H_2_O, Reflux, 4h, 55–94% Over Two Steps; (iii) EtOH, HCl 37%_cat_, Aldehyde, Room Temp., 4h, 59–75%

**Figure 4:**
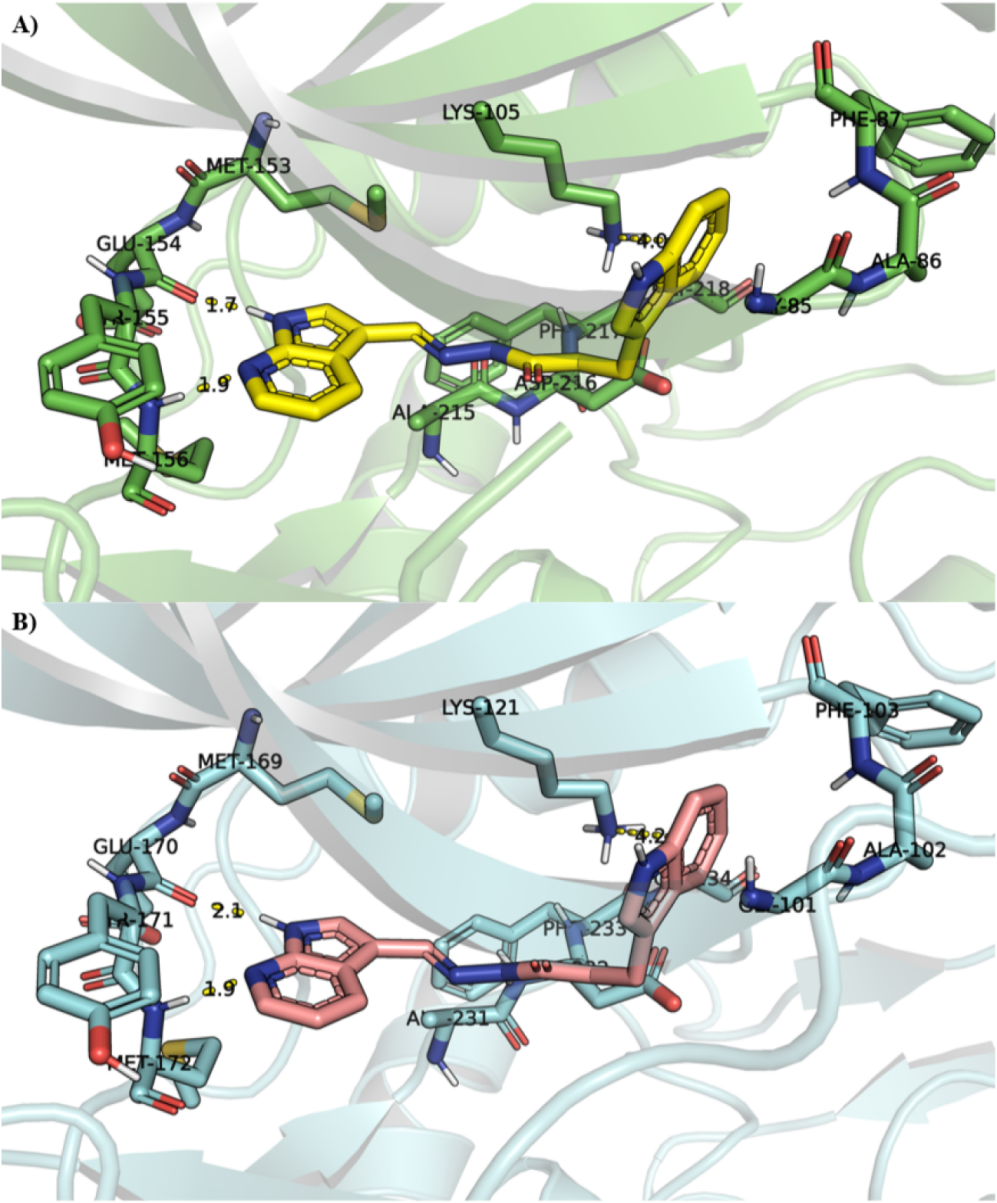
A) Interaction mode of **12** (carbons in yellow) in ROCK1 (PDB 6E9W)^11^ calculated by docking studies. B) Interaction mode of **12** (carbons in pink) in ROCK2 (PDB 7JNT)^15^ calculated by docking studies.

**Table 1:**
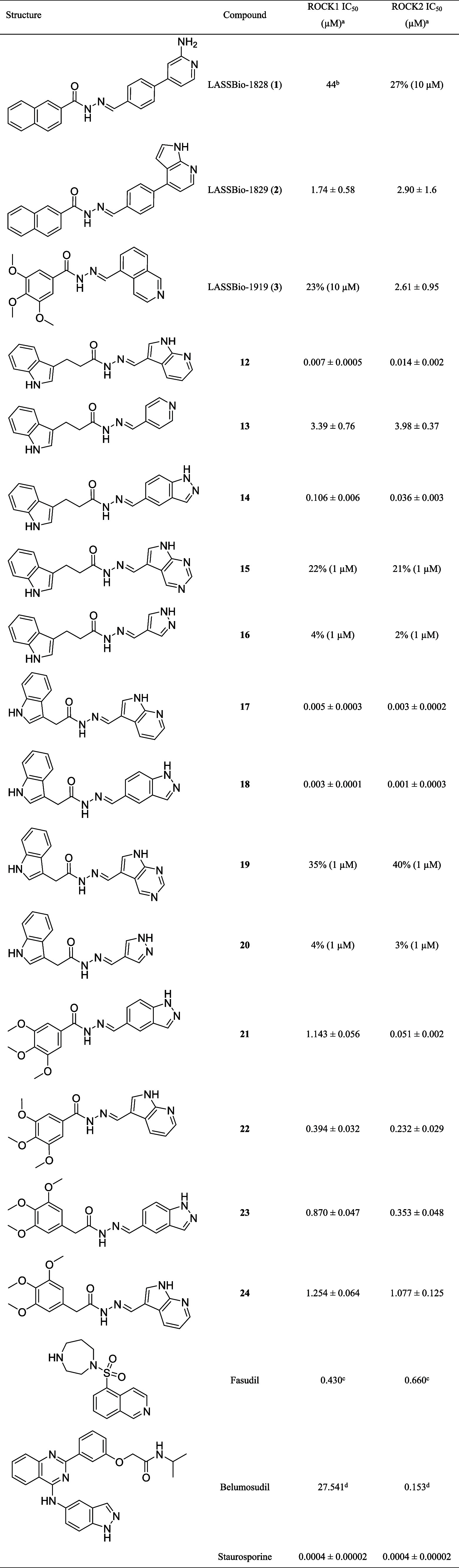
IC_50_ Data for ROCK Inhibition of a Congeneric Series of *N*-Acylhydrazone Derivatives^a^

a Studies performed by Reaction Biology Corp., Malvern, PA/USA (Study number: 20240122-UFRJ-CF-KP; 20241218-UFRJ-PP-Mix; 20250114-UFRJ-PP-Mix). Compounds were tested in 10-dose IC_50_ mode with 3-fold serial dilutions.

b IC_50_ was estimated by extrapolation from the dose–response curve beyond the tested concentration range.

c IC_50_ data retrieved from a previous study.^41^

d IC_50_ data retrieved from a previous study.^4^

Next, we design a new congeneric series of NAH derivatives using **12** as a prototype. We applied a combination of homologation^28^ and bioisosteric replacement^29^ strategies in order to evaluate the linker size and the effect of replacing the 7-azaindole pharmacophore core with other bicyclic heteroaromatic rings, such as indazole, 5,7-azaindole, and pyrazole, maintaining the acceptor/donor hydrogen bond features, giving rise to compounds **14**-**20**. In addition, four analogues were designed (**21**-**24**) based on molecular hybridization,^30^ in which the common terminal indole moiety was replaced by a trimethoxyphenyl from LASSBio-1919 (**3**). This was done in order to analyze the trimethoxyphenyl group in depth, as an auxophoric group capable of generating ROCK2 selectivity over ROCK1.

### Chemical Synthesis

The designed molecules were obtained through a linear synthetic route shown in Scheme 1. Initially, the commercially available corresponding carboxylic acids (**25**, **26**, **29** and **30**) were converted to their corresponding methyl ester derivatives using Fischer esterification conditions. Next, the esters were converted to their corresponding hydrazide derivatives (**27**, **28**, **31** and **32**) by carbonyl substitution reaction in the presence of hydrazine hydrate. Finally, the aimed compounds (**12**-**24**) were obtained using condensation reactions with the corresponding aldehydes, catalyzed in an acidic medium.

The characterization of the synthesized NAH derivatives was conducted using^1^H and ^13^C Nuclear Magnetic Resonance (NMR) spectroscopy, high pressure liquid chromatography (HPLC) and high-resolution mass spectrometry (HRMS). The NMR spectra of all compounds bearing one or two methylene units between the carbonyl group and the NAH moiety displayed duplicated signals, as illustrated in Figures S1 and S3, which shows the ^1^H and ^13^C spectrum of compound **18**, respectively. This duplication initially raised the question of a possible mixture of diastereomers or conformers. ^1^H NMR spectra acquired at 80 °C showed coalescence of duplicated signals (Figure S2), confirming that the duplication arises from conformers. Moreover, this is likely a consequence of *syn-* and *anti*-rotamers around the NAH amide σC–N bond. Potential energy surface (PES) scans using the ωB97-XD/6–311+G­(d,p) hybrid functional for evaluation of the amide σC–N bond of **18** and its superior homologue **14** revealed an energy barrier of approximately 16 kcal·mol^–1^ at room temperature in polar solvent, consistent with the presence of a slow dynamic equilibrium between rotamers (Figure S4), as already reported in previous studies of structure–property relationship of NAH derivatives.^31–33^ Moreover, the (*E*) configuration of the NAH moiety is further supported by the chemical shift of the imine proton and by previous studies on related bioactive NAH.^31,34–38^

### SAR of the Congeneric Series of Hydrazones for ROCK Inhibition

The evaluation of NAH derivatives against ROCK1 and ROCK2 revealed a broad spectrum of activity, ranging from low micromolar to low nanomolar potency, along with distinct selectivity patterns. Among the derivatives with a two-methylene linker (**12**–**16**), the best hinge-binding scaffolds were identified as the 7-azaindole (**12**), as previously noted, and the indazole (**14**), which exhibited IC_50_ values of 0.106 μM for ROCK1 and 0.036 μM for ROCK2. In contrast, replacing the 7-azaindole with a 5,7-azaindole (**15**) or simplifying the indazole to a pyrazole ring (**16**) produced inactive compounds, highlighting the importance of these specific scaffolds. Reducing the linker length to a single methylene generated the inferior homologues **17**–**20**, among which the 7-azaindole derivative **17** (IC_50_ = 0.005 μM for ROCK1 and 0.003 μM for ROCK2) and the indazole derivative **18** (IC_50_ = 0.003 μM for ROCK1 and 0.001 μM for ROCK2) emerged as the most potent compounds of the series. Comparison of **17** and **18** with their superior homologues, i.e., **12** and **14**, clearly demonstrates that the one-methylene linker provides the optimal length to engage the ROCK1 and ROCK2 affinity pockets, likely by better positioning the scaffold to establish a cation−π interaction with the catalytic lysine. Conversely, compounds **19** and **20** were inactive against both kinases at 1 μM. The trimethoxyphenyl derivatives (**21**–**24**) also provided insightful results. Compound **21** displayed remarkable ROCK2 selectivity, with an IC_50_ of 0.051 μM for ROCK2 and 1.143 μM for ROCK1, showing 21-fold selectivity for ROCK2 over ROCK1. However, substituting the indazole (**21**) with a 7-azaindole (**22**) abolished this selectivity, yielding similar potencies against both ROCK1 (IC_50_ = 0.394 μM) and ROCK2 (IC_50_ = 0.232 μM). Increasing the linker length by one methylene while maintaining the indazole core afforded compound **23** (IC_50_ = 0.870 μM for ROCK1 and 0.353 μM for ROCK2), which showed reduced ROCK2 potency, slightly improved ROCK1 inhibition, and overall loss of selectivity in comparison with **21**. Similarly, extending the linker in compound **22** gave rise to compound **24** (IC_50_ = 1.254 μM for ROCK1 and 1.077 μM for ROCK2), resulting in decreased potency for both targets. These findings establish a clear structure–activity relationship within the NAH series. Derivatives **12**, **17**, and **18** emerged as the most potent inhibitors, achieving balanced ROCK1 and ROCK2 inhibition at low nanomolar concentrations, while compound **21** stood out by exhibiting 21-fold selectivity for ROCK2. Achieving ROCK2 selectivity is particularly challenging, given that ROCK1 and ROCK2 share more than 90% sequence identity within their kinase domains.^39^ Although a recent study suggested that ROCK2 selectivity may arise from interactions with a hydrophobic pocket accessible only in ROCK2 as a result of a DFG-out conformation,^40^ this conformation is induced by a shift in the α*C*-helix that can occur in ROCK2 but is hindered in ROCK1 due to the residue difference L210 in ROCK2 versus F194 in ROCK1^41^ (for details, see Figure S5 in the Supporting Information). In this way, ROCK2-selective inhibitors function as type 2 kinase inhibitors.^14^ Taken together, our results underscore the NAH scaffold as a promising framework for the development of potent and potentially isoform-selective ROCK inhibitors.

To obtain an initial overview of selectivity, compounds **18** and **21** were profiled at 500 nM against a representative panel of kinases structurally related to ROCK—IKKβ/IKBKB, JAK1, MLCK/MYLK, PKAcβ, PKCα, and PKG1α (Table 2). Importantly, **18** is substantially more potent on ROCK2 (IC_50_ = 1 nM) than **21** (IC_50_ = 51 nM); therefore, screening both compounds at a single fixed concentration (500 nM) means that **18** was evaluated at ∼500-fold its ROCK2 IC_50_, whereas **21** was evaluated at ∼10-fold. This difference in on-target potency can influence the apparent selectivity margin across a small panel when only one concentration is used.

**Table 2:** Comparative Inhibition of Representative Kinases by Compounds **18** and **21**

	Kinase Inhibition (%) at 500 nM^a^	
	**18**	**21**
IKKβ/IKBKB	4.0	2.7
JAK1	5.3	4.2
MLCK/MYLK	15.4	8.1
PKAcβ	94.0	4.4
PKCα	6.5	2.3
PKG1α	98.9	32.9

a Studies performed by Reaction Biology Corp., Malvern, PA/USA (Study number: 20251002-UFRJ-PP-Mix-RV02).

**18** displayed potent off-target inhibition of PKAcβ (94%) and PKG1α (98.9%), indicating a limited selectivity within the AGC subfamily. In contrast, **21** exhibited minimal inhibition of all tested kinases (<10%) except for PKG1α (32.9%), suggesting a markedly improved selectivity profile relative to **18**. These results reinforce that the indazole–trimethoxyphenyl hybrid structure of **21** confers superior discrimination against the tested kinases, potentially linked to its ability to stabilize the DFG-out conformation of ROCK2 rather than interacting promiscuously with other kinases. Additional profiling across a broader kinase panel would further elucidate the selectivity landscape of this chemotype.

### Docking and Molecular Dynamics Simulations of Compounds 18 and 21

Compounds **18** and **21** were investigated for their interaction modes within ROCK1 (PDB: 6E9W ^*DFG‑in*^)^11^ and ROCK2 (PDB: 7JNT ^*DFG‑in*^ and 8GDS^*DFG‑out*^).^15,40^ Two ROCK2 crystal structures were employed, since 8GDS presents the open hydrophobic pocket caused by a shift of the α*C*-helix (DFG-out conformation). The aim was to characterize the binding modes of both compounds and, through molecular dynamics simulations, to explore the molecular basis underlying the 21-fold selectivity of **21** for ROCK2 over ROCK1. We anticipated that **21** would exhibit a better fit in the 8GDS structure compared to 7JNT and 6E9W.

The binding poses of **18** and **21** in 6E9W and 7JNT followed the expected pattern: both compounds established bidentate hydrogen bonds with the hinge region, interacting with the backbone of the corresponding glutamate and methionine residues (Figure 5A–D). In addition to hinge anchoring, the linker length relative to compound **12** is central for the alignment between the hinge-binding region and the terminal aromatic system in this series. **12** (two methylene units) is already capable of establishing a Lys-centered cation−π interaction within the affinity pocket; however, shortening the spacer to one methylene unit in **18** optimizes the geometry of this interaction and improves the overall fit of the terminal aromatic group toward the affinity pocket. In contrast, removing the methylene spacer in **21** (direct NAH connection) shortens the reach and is not expected to sustain the same cation−π interaction, which is consistent with the ROCK2 potency differences (Table 1). Moreover, unlike derivative **12**, the indazole nucleus oriented the carbonyl oxygen of the NAH moiety toward the catalytic lysine, forming a stable hydrogen bond. This interaction was observed for both **18** and **21** in ROCK1 (6E9W) and ROCK2 (7JNT). Moreover, compound **18** was capable of forming cation−π interactions within the affinity pocket framed by the catalytic lysine and the G-loop.

**Figure 5:**
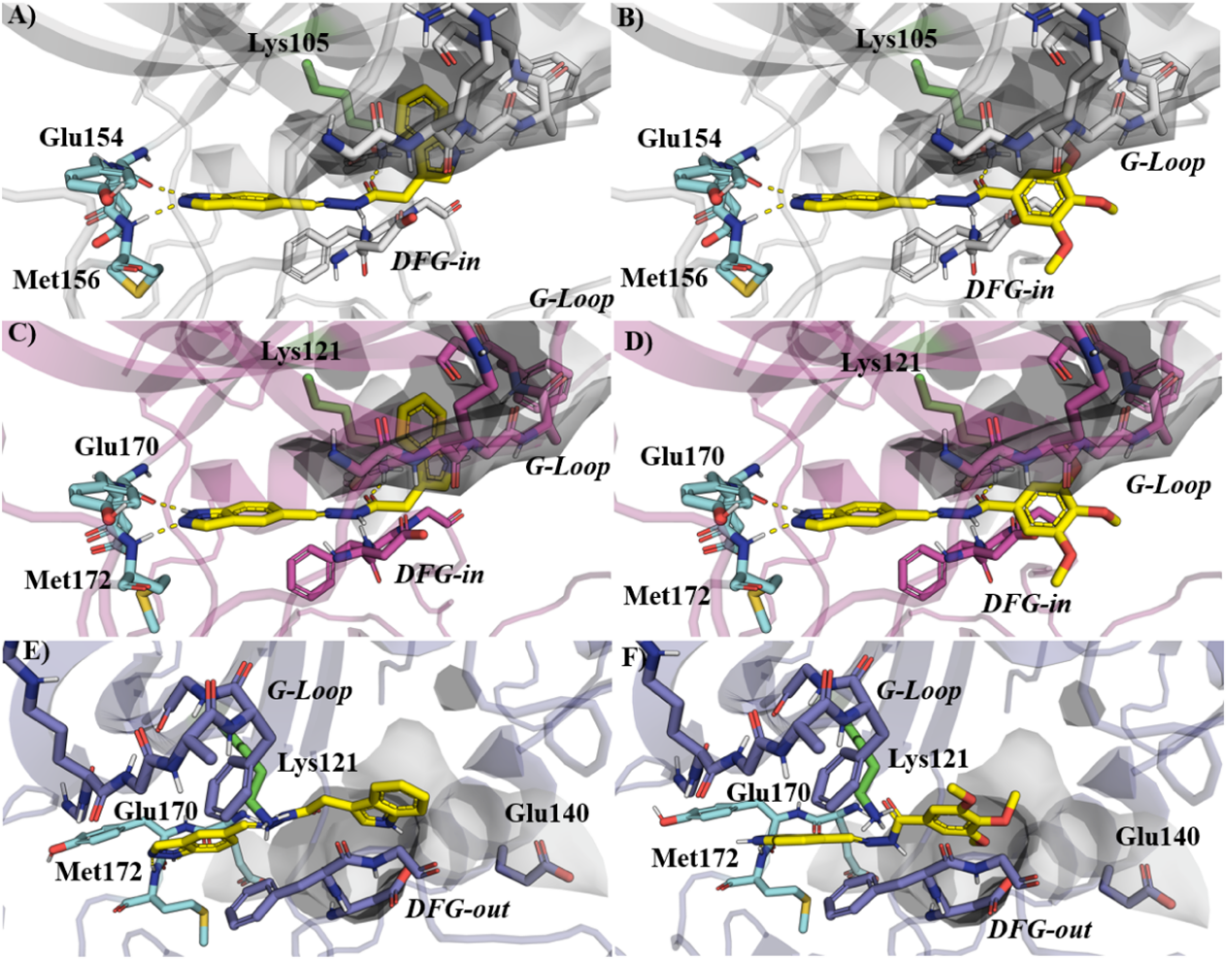
Binding modes of compounds **18** and **21** within ROCK1 and ROCK2 as predicted by molecular docking. Panels A and B show the binding poses of **18** and **21** in the ROCK1 crystal structure (PDB: 6E9W), while panels C and D depict their interactions in the ROCK2 crystal structure (PDB: 7JNT). Panels E and F illustrate the binding modes of **18** and **21** in the ROCK2 structure with the open allosteric pocket (PDB: 8GDS). Ligand carbon atoms are shown in yellow. Carbons of residues from the hinge region are shown in cyan.

It is noteworthy that the 8GDS crystal of ROCK2 presents a significant conformational rearrangement, not only involving the αC-helix and the DFG motif but also the catalytic lysine, as illustrated in Figure S5. In this conformation, the catalytic lysine is displaced forward, assisting in the orientation of the cocrystallized ligand toward the open hydrophobic pocket, while simultaneously closing the affinity pocket previously formed between the lysine and the G-loop. At first glance, neither **18** nor **21** displayed an optimal fit in this structure compared to 7JNT (Figure 5E,F). For **18**, the hydrogen bonds appeared less directional, whereas **21** adopted a monodentate hinge interaction, forming a single hydrogen bond with Met172. Due to the displacement of the catalytic lysine, hydrogen bonding with this residue was lost, however, the terminal aromatic subunits of both compounds shifted to occupy the hydrophobic pocket.

These docking results provided the structural basis for subsequent molecular dynamics simulations aimed at evaluating the stability of the complexes and elucidating the structural determinants responsible for the enhanced ROCK2 selectivity of compound **21**.

To further investigate the structural determinants underlying the differential inhibition of ROCK1 and ROCK2, molecular dynamics (MD) simulations were carried out for **18** and **21** complexed with ROCK1 (6E9W^*DFG‑in*^), ROCK2 (7JNT^*DFG‑in*^), and ROCK2 (8GDS^*DFG‑out*^). The simulations provided insights into the conformational stability of the complexes and into the ability of each ligand to accommodate and stabilize the distinct kinase conformations.

Overall RMSD profiles (Figure 6A–C) indicated that all systems reached equilibration during the simulation time. In ROCK1 (6E9W), the total RMSD values stabilized between 3–4 Å for the ligand-bound systems and 2–3 Å for the apo form, suggesting that ligand binding did not induce significant global rearrangements. Similar behavior was observed for ROCK2 (7JNT), with RMSD values between 4–5 Å for both 18 and 21, and 2–3 Å for the apo enzyme. In the 8GDS structure (ROCK2^*DFG‑out*^), all complexes displayed slightly higher fluctuations (4–5 Å), reflecting the intrinsic flexibility of this open conformation. RMSF analysis (Figure 6D–F) further confirmed that ligand binding did not significantly alter the mobility pattern of key secondary-structure elements, indicating that conformational changes were localized rather than global.

**Figure 6:**
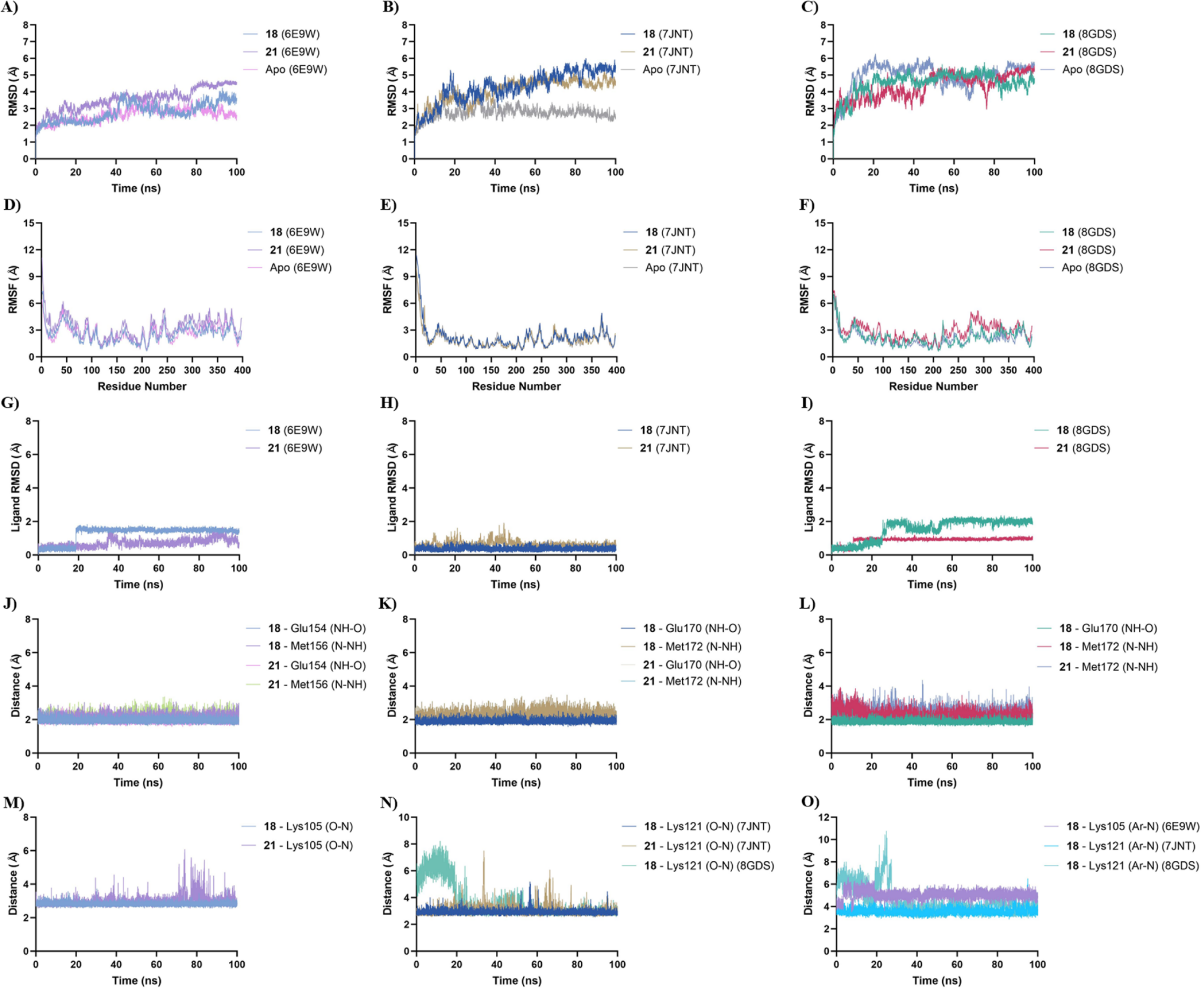
MD simulation data for compounds **18** and **21** in complex with ROCK1 (PDB ID: 6E9W *^DFG‑in^*), ROCK2 (PDB ID: 7JNT *^DFG‑in^*), and ROCK2 (PDB ID: 8GDS *^DFG‑out^*). (A–C) Backbone RMSD plots for apo and ligand-bound systems of 6E9W (A), 7JNT (B), and 8GDS (C). (D–F) RMSF profiles for 6E9W (D), 7JNT (E), and 8GDS (F). (G–I) Ligand RMSD plots for **18** and **21** in 6E9W (G), 7JNT (H), and 8GDS (I). (J–L) Time evolution of hydrogen-bond distances between the ligands and hinge residues for 6E9W (J), 7JNT (K), and 8GDS (L). (M–N) Distance between the carbonyl oxygen of the *N*-acylhydrazone moiety and the *ε*-amino nitrogen of the catalytic lysine in ROCK1 (M) and ROCK2 (N). (O) Distance between the terminal indole ring of **18** and the catalytic lysine, corresponding to the cation−π interaction.

Ligand RMSD profiles (Figure 6G–I) showed remarkable stability, remaining below 2 Å throughout the simulations, except for compound **18** in 8GDS, which required approximately 50 ns to reach equilibration. This higher fluctuation was accompanied by a conformational transition from the DFG-out to the DFG-in state, suggesting that **18** does not efficiently stabilize the open hydrophobic pocket characteristic of the DFG-out conformation. In contrast, **21** maintained the DFG-out conformation throughout the trajectory, indicating its greater structural compatibility with this ROCK2-specific state. This observation provides a dynamic explanation for the experimentally observed 21-fold selectivity of compound **21** toward ROCK2.

Hydrogen bond analysis with the hinge residues Glu154/Met156 (ROCK1) and Glu170/Met172 (ROCK2) confirmed the persistence of key interactions across all systems (Figure 6J–L), with distances consistently around 2 Å. In agreement with the docking results, **21** formed a monodentate hydrogen bond with Met172 in the DFG-out structure (8GDS), while maintaining a stable orientation within the binding pocket.

The distance between the carbonyl oxygen of the *N*-acylhydrazone moiety and the catalytic lysine (Lys105 in ROCK1; Lys121 in ROCK2) remained close to 3 Å in both ROCK1 and ROCK2 (7JNT) complexes (Figure 6M,N). However, in the ROCK2 (8GDS) complex with **18**, the distance initially increased to approximately 8 Å during the first 20 ns and then gradually decreased and stabilized near 3 Å (Figure 6N). This shift in distance reflects the conformational rearrangement of the activation loop, marking the transition from the DFG-out to the DFG-in state.

The cation−π interactions involving the indole moiety of **18** and the catalytic lysine were also analyzed (Figure 6O). In ROCK1 (6E9W) and ROCK2 (7JNT), these distances remained stable around 5 Å and 4 Å, respectively, corroborating the presence of persistent electrostatic interactions within the affinity pocket. Conversely, in the DFG-out structure (8GDS), the distance initially increased to 10 Å before stabilizing near 4 Å after 20 ns, further reflecting the structural rearrangement of the catalytic lysine during the conformational transition.

Taken together, the MD results demonstrate that **21** not only fits well within the DFG-out conformation of ROCK2 but also stabilizes it throughout the simulation, maintaining productive hinge interactions and consistent orientation of its *N*-acylhydrazone core. In contrast, **18** fails to preserve the open hydrophobic pocket, favoring the return to a DFG-in–like arrangement. These findings support the hypothesis that the selectivity of **21** for ROCK2 arises from its ability to stabilize and exploit the DFG-out conformation, a structural feature that is energetically disfavored or sterically hindered in ROCK1.

### PAMPA Assay

Prior to cellular evaluation, compounds **12**, **17**, **18**, and **21**, along with the reference inhibitors fasudil and belumosudil, were assessed for permeability using the Parallel Artificial Membrane Permeability Assay (PAMPA). In the PAMPA-GIT model, fasudil (*Pe* = 3.575 × 10^–6^ cm/s) and **21** (*Pe* = 5.562 × 10^–6^ cm/s) were classified as highly permeable, corresponding to predicted absorbed fractions of 74% and 88%, respectively. The remaining compounds were not soluble under the experimental conditions that mimic the gastrointestinal barrier. In the PAMPA-BBB model, fasudil (*Pe* = 1.364 × 10^–6^ cm/s) and **21** (*Pe* = 0.537 × 10^–6^ cm/s) were classified as nonpermeable (CNS−), while **12** (*Pe* = 2.453 × 10^–6^ cm/s) and **17** (*Pe* = 2.614 × 10^–6^ cm/s) showed uncertain permeability (CNS±). Belumosudil and **18** were insoluble under the experimental conditions, which mimic the blood-brain barrier.

### Cell Viability Assay

Compounds **12**, **17**, **18**, and **21** were selected for in vitro functional evaluation to confirm ROCK engagement at the cellular level. Initially, cell viability was assessed after 24 h exposure to tested compounds at a concentration of 30 μM, using 1% DMSO as the vehicle. Fasudil and belumosudil were included as reference inhibitors. Results were normalized to the 1% DMSO control (Figure 7). At the tested concentration, all compounds reduced cell viability by less than 30%, with **17** showing the strongest effect (28.8% inhibition).

**Figure 7:**
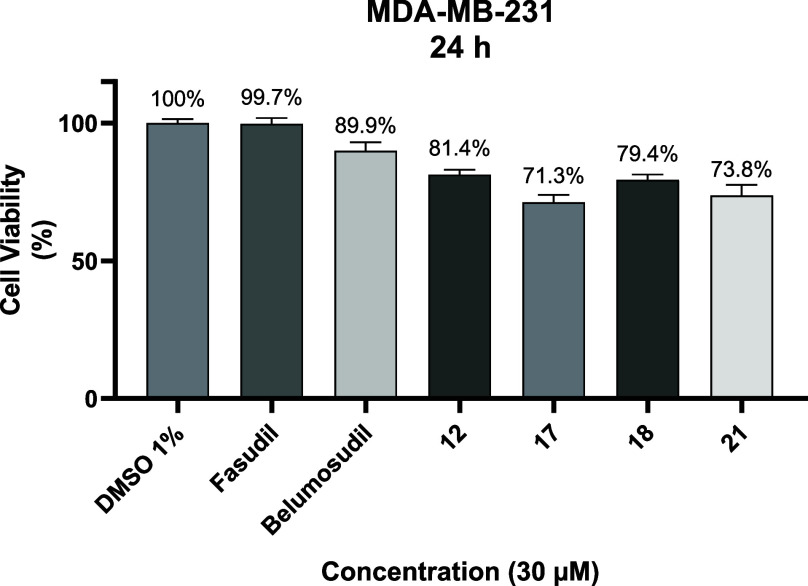
Cell viability analysis of MDA-MB-231 cells after 24 h of incubation in the presence of the controls (fasudil and belumosudil) and compounds **12**, **17**, **18**, and **21** at 30 μM. Data presented as mean ± SD of three different experiments with 95% confidence interval (*p* < 0.05).

### Wound Healing Assay

The effects of the selected compounds on cell migration were evaluated using a wound healing (scratch) assay with the MDA-MB-231 triple-negative breast cancer cell line. This cell line is known for its high aggressiveness and metastatic potential and exhibits overexpression of both ROCK isoforms, with ROCK2 levels exceeding those of ROCK1.^5^ Considering that cell migration and invasion are crucial processes in cancer metastasis, and that the Rho/ROCK signaling pathway plays a central role in their regulation,^42^ we examined the effects of molecules developed in our research group as potential ROCK1/2 inhibitors on this cellular model.

Cells were treated with four concentrations (0.1, 10, 30, and 100 μM) of the test compounds **12**, **17**, **18**, and **21**, as well as the reference inhibitors fasudil and belumosudil, using 1% DMSO as a vehicle control. **12** and its inferior homologue **17**, quantification of wound closure was not possible at 100 μM due to compound precipitation (Figure 8A,B, respectively). For **12**, precipitation was also observed at 30 μM. As shown in Figure 8, both compounds inhibited migration from 10 μM. **12** showed similar inhibition at 10 and 30 μM, whereas **17** demonstrated enhanced inhibition at 30 μM, likely due to improved solubility at this concentration. For both compounds, changes in cell morphology became apparent above 10 μM (Figures S7 and S8, Supporting Information) compared with the DMSO control.

**Figure 8:**
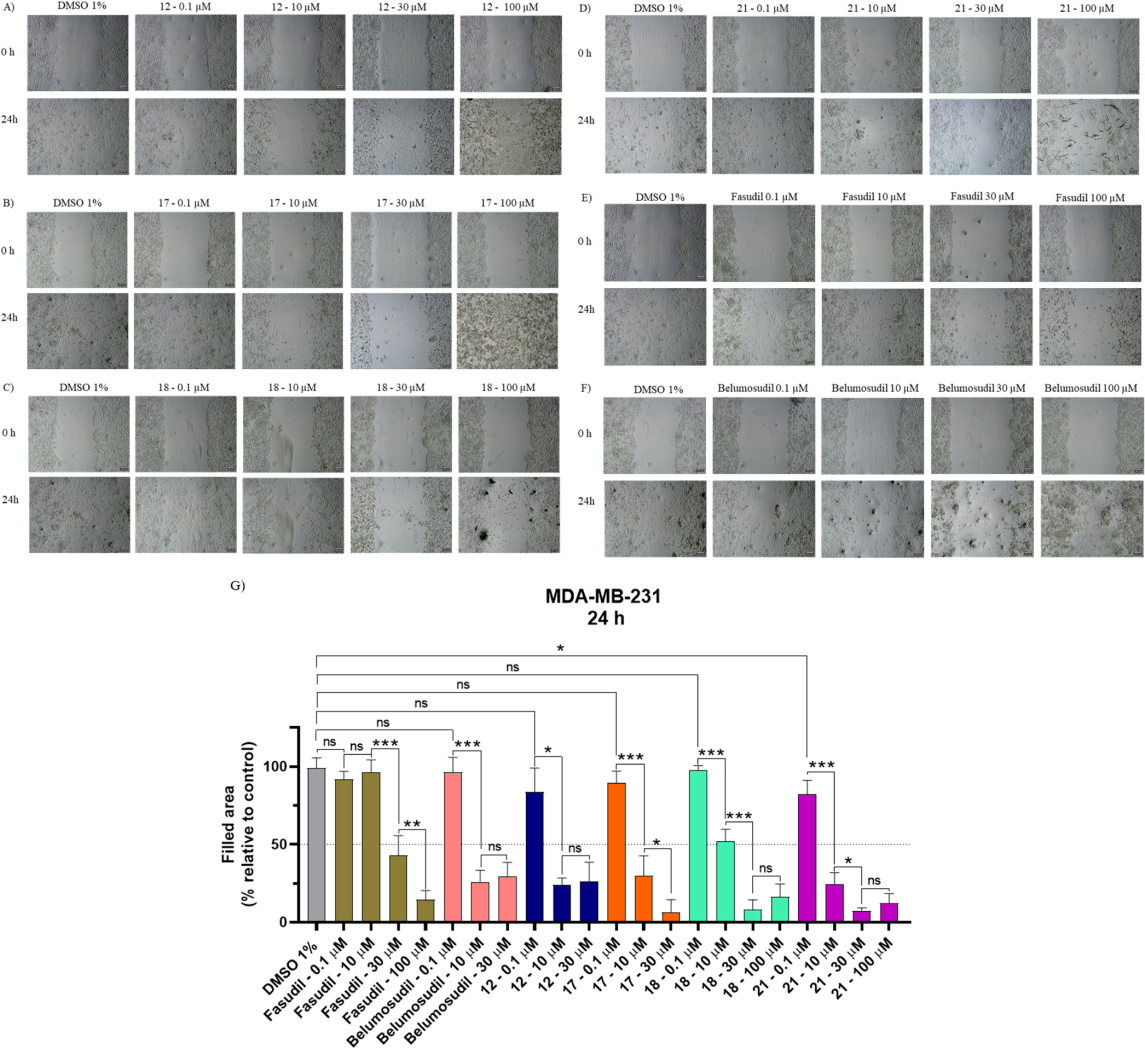
Wound healing assay in MDA-MB-231 cells. Representative micrographs showing cell migration at 0 h and after 24 h exposure to (A) **12**, (B) **17**, (C) **18**, (D) **21**, (E) fasudil, and (F) belumosudil. Scale bars: 100 μm. (G) Quantitative analysis of cell migration expressed as the percentage of filled wound area relative to the 1% DMSO control. Data are presented as mean ± SD from three independent experiments, with 95% confidence intervals (*p* < 0.05).

According to Figure 8C,G, **18** reduced wound closure to less than 50% at 10 μM and to below 25% at 30 μM, showing similar inhibition at 100 μM. Although precipitation occurred at 100 μM, no visible precipitate was detected at 30 μM. Morphological alterations of the cells were evident from 30 μM onward (Figure S9, Supporting Information). **21** exhibited a pronounced effect at lower concentrations: as early as 0.1 μM, a significant reduction in migration was observed (*p* < 0.05) compared with the control. At 10 μM, the wound closure area was reduced by ≤25% (Figure 8D,G). At 30 μM, fewer crystals were visible in the well, whereas at 100 μM, compound precipitation was clearly observed (Figure S10, Supporting Information). Morphological changes were also detected at concentrations above 10 μM.

As shown in Figure 8E,G, fasudil reduced cell migration by approximately 50% after 24 h at 30 μM. Microscopic analysis revealed that at 30 μM, the wound area remained largely unfilled compared with lower concentrations, and at 100 μM, wound closure was minimal. In addition, morphological changes in the cells were evident at 100 μM (Figure S11), suggesting that cell viability may be compromised at higher concentrations.

In Figure 8F,G, the results for belumosudil are shown. Migration inhibition was observed from 10 μM onward, with the filled area reduced to less than 50% at both 10 and 30 μM, with no significant difference between these two concentrations. At 100 μM, the assay could not be quantified due to poor solubility of the compound, and amorphous precipitates were already visible at 30 μM (Figure S12). Although both fasudil and belumosudil act as ROCK1/2 inhibitors, belumosudil inhibited migration at lower concentrations. This behavior aligns with its reported selectivity for ROCK2, whereas fasudil displays minimal isoform preference (>2-fold), despite showing similar potency toward ROCK2.^43^ Differences in cell permeability and compound solubility may also contribute to these results.

Notwithstanding, because our selectivity assessment was restricted to a limited set of structurally related kinases, we cannot exclude that part of the migration phenotype—especially at higher concentrations where morphological alterations were observed—may involve off-target contributions. While the overall behavior is consistent with ROCK pathway modulation and aligns with the effects of the reference inhibitors (fasudil and, particularly, the ROCK2-selective belumosudil), a broader kinase profiling panel and orthogonal target-engagement readouts in cells will be important in future studies to more conclusively disentangle on-target ROCK inhibition from potential off-target mechanisms.

Taken together, the four identified NAH derivatives (**12**, **17**, **18**, and **21**), showed stronger inhibition of cell migration than fasudil and comparable effects to belumosudil in the MDA-MB-231 cell line. These findings support their potential as promising leads to modulate the Rho/ROCK signaling pathway in highly invasive cancers, while highlighting the need for expanded selectivity profiling and orthogonal cellular target-engagement studies to further substantiate the mechanism.

## Conclusion

In this study, a rational and integrative strategy combining structure-based virtual screening, structure-based de novo design, molecular modeling, and experimental validation enabled the discovery of potent and selective ROCK inhibitors within the NAH chemical space. Starting from the LASSBio Chemical Library, the approach led to the identification of LASSBio-1829 (**2**) and LASSBio-1919 (**3**) as initial hits, which guided the structure-based de novo design approach of new derivatives featuring optimized hinge binders and flexible linkers, followed by experimental validation of two selected NAH derivatives and SAR exploration. Among the synthesized compounds, derivatives **12**, **17**, and **18** exhibited balanced low nanomolar inhibition of both ROCK1 and ROCK2, whereas **21** demonstrated remarkable ROCK2 selectivity (21-fold), a rare and therapeutically valuable property. In addition, we acknowledge that a certain degree of structural similarity at the hinge-anchoring region is a general feature of ROCK inhibitors, as can be observed in Figure 1, reflecting the conserved hydrogen-bonding requirements for productive ATP-site recognition.

Assays against structurally related kinases revealed that **21** inhibited less than 10% of the evaluated off-targets at 500 nM—except for PKG1α (32.9%)—while **18** strongly inhibited PKAcβ (94%) and PKG1α (98.9%), indicating that the indazole–trimethoxyphenyl hybrid scaffold of **21** confers substantial selectivity for ROCK2 over other related kinases.

Molecular docking and molecular dynamics simulations revealed that **21** preferentially stabilizes the DFG-out conformation of ROCK2, providing a mechanistic explanation for its isoform selectivity. In the PAMPA assay, only **21** showed measurable passive permeability under our experimental conditions, supporting its biopharmaceutical profile among the tested derivatives. In cell-based assays, the evaluated compounds significantly reduced migration of MDA-MB-231 triple-negative breast cancer cells at micromolar concentrations, providing a functional phenotype consistent with modulation of migration-related pathways. However, these functional assays do not constitute direct evidence of cellular ROCK1/2 target engagement, and off-target contributions cannot be excluded given the limited kinase selectivity profiling performed to date. Future studies incorporating orthogonal cellular target-engagement readouts and broader kinase profiling will be important to more conclusively attribute the observed phenotype to on-target ROCK inhibition.

Overall, our findings establish *N*-acylhydrazone as a versatile and synthetically accessible scaffold for the development of next-generation ROCK inhibitors, highlighting key structural determinants for potency and isoform selectivity. These results not only expand the chemical space of ROCK modulators but also open new opportunities for the design of antimetastatic and antifibrotic agents targeting ROCK2 with improved efficacy and safety.

Importantly, we acknowledge a potential limitation of the *N*-acylhydrazone scaffold related to chemical stability. Acylhydrazones can be susceptible to hydrolysis under aqueous/physiological conditions, which may regenerate the corresponding aldehyde and hydrazide, depending on factors such as pH, substituent electronics, and the local environment. While the present work focuses on potency, selectivity, and cellular activity, future studies will explicitly address this liability through systematic stability profiling in buffered media and biological matrices (e.g., plasma) and by monitoring potential degradation products. Such data will guide further optimization efforts, including stabilizing structural modifications or the exploration of bioisosteric alternatives when needed.

## Experimental Session

### Chemistry

#### General Information

The melting points of the compounds were determined using a Quimis 340 apparatus and are corrected. ^1^H NMR spectra were determined in dimethyl sulfoxide and the solvent was used as an internal standard using a Varian 500-MR at 500 MHz or a Varian 400-MR at 400 MHz. ^13^C NMR spectra were resolved using the same solvent and spectrometers at 125 or 100 MHz. The progress of all reactions was monitored through thin-layer chromatography performed on 2.0 × 6.0 cm aluminum sheets precoated with silica gel 60 (HF-254, Merck) to a thickness of 0.25 mm. The developed chromatograms were analyzed under ultraviolet light (254–365 nm). The reagents and solvents were purchased from commercial suppliers and used as received. Analytical HPLC was performed for compound purity determinations using a Shimadzu LC- 20AD with a Kromasil 10 C18 column (4.6 mm × 259 mm) and a Shimadzu SPD-M20A detector. The solvent system used for the HPLC analyses was (A) methanol/water at 7:3 or (B) acetonitrile/water 5:5. The isocratic HPLC mode was used, and the flow rate was 1.0 mL/min. The purity of the compounds was higher than 95%. High-resolution mass spectrometric analyses were performed on a Q Exactive hybrid quadrupole–Orbitrap mass spectrometer (Thermo Fisher Scientific, Bremen, Germany) equipped with an electrospray ionization (ESI) source. Samples were prepared by adding methanol with 0.1% formic acid, resulting in a final concentration of 10 μg/mL. The samples were analyzed by direct infusion at a flow rate of 10 μL/min. Spectra were acquired over the *m*/*z* range 100–1500 at a resolving power of 70,000 (fwhm at *m*/*z* = 200).

### Synthesis of the *N*-Acylhydrazone Derivatives 1, 2 and 3

Compounds **1**, **2**, and **3** were synthesized as previously described.^24–26^ The structures of all compounds were confirmed by ^1^H NMR spectroscopy, and the spectra were consistent with the data previously reported. HPLC analyses indicated that all compounds exhibited purity higher than 95%.

### Procedure for the Synthesis of Hydrazide Intermediates (27, 28, 31 and 32)

The corresponding carboxylic acid (1.0 equiv., 2 g) was dissolved in 200 mL of methanol (MeOH) in a 500 mL round-bottom flask. Subsequently, 1.0 mL of a 1 M H_2_SO_4_ solution in MeOH was added, and the reaction mixture was refluxed for 12 h under magnetic stirring. After completion, hydrazine monohydrate (10.0 equiv., 100%) was added to the same flask. The reaction mixture was then refluxed for an additional 2 h under magnetic stirring. The solvent was removed under reduced pressure and the resulting solid was resuspended in *n*-hexane, filtered under vacuum, and washed with diethyl ether to afford the desired hydrazide.

#### 2-(1H-Indol-3-yl)­acetohydrazide (27)

The title compound was obtained as a white solid in 55% yield (MP: 125–128 °C). ^1^H NMR (500 MHz, DMSO-*d*_6_) δ 10.64 (s, 1H), 8.82 (s, 1H), 7.57 (d, *J* = 7.9 Hz, 1H), 7.34 (d, *J* = 8.1 Hz, 1H), 7.17 (d, *J* = 2.2 Hz, 1H), 7.09 – 7.04 (m, 1H), 7.00 – 6.96 (m, 1H), 4.12 (s, 2H), 3.49 (s, 2H).

#### 3-(1H-Indol-3-yl)­propanehydrazide (28)

The title compound was obtained as a white solid in 59% yield (MP: 118–121 °C). ^1^H NMR (500 MHz, DMSO-*d*_6_) δ 10.52 (s, 1H), 8.76 (s, 1H), 7.52 (d, *J* = 7.9 Hz, 1H), 7.33 (d, *J* = 8.1 Hz, 1H), 7.08 – 7.04 (m, 2H), 6.99 – 6.95 (m, 1H), 4.09 (s, 2H), 2.95 (t, *J* = 7.7 Hz, 2H), 2.42 (t, *J* = 7.7 Hz, 2H).

#### 3,4,5-Trimethoxybenzohydrazide (31)

The title compound was obtained as a white solid in 93% yield (MP: 160–163 °C). ^1^H NMR (500 MHz, DMSO-*d*_6_) δ 9.50 (s, 1H), 7.17 (s, 2H), 4.37 (s, 2H), 3.83 (s, 6H), 3.74 (s, 3H).

#### 2-(3,4,5-Trimethoxyphenyl)­acetohydrazide (32)

The title compound was obtained as a white solid in 94% yield (MP: 120–123 °C). ^1^H NMR (500 MHz, DMSO-*d*_6_) δ 8.90 (s, 1H), 6.58 (s, 2H), 4.13 (s, 2H), 3.77 (s, 6H), 3.67 (s, 3H), 3.30 (s, 2H).

#### Procedure for the Synthesis of the *N*-Acylhydrazone Derivatives 12–24

The corresponding hydrazide (1.0 mmol) was dissolved in 10 mL of absolute ethanol in a 50 mL round-bottom flask. The appropriate aldehyde (1.0 mmol) and two drops of 37% HCl solution were then added, and the reaction mixture was refluxed for 4 h. Reaction progress was monitored by TLC (eluent: dichloromethane:methanol, 9.5:0.5 v/v). Upon completion, the reaction mixture was cooled to 0 °C, and the resulting solid was collected by vacuum filtration. When necessary, the crude products were further purified by recrystallization from ethanol/water.

#### (E)-*N*′-((1H-Pyrrolo­[2,3-*b*]­pyridin-3-yl)­methylene)-3-(1H-Indol-3-yl)­propanehydrazide (**12**)

The title compound was obtained as a yellow solid in 70% yield (MP: 245–248 °C). ^1^H NMR (500 MHz, DMSO-*d*_6_) δ 12.04 (s, 1H), 11.15, 11.06 (s, 1H), 10.80 (s, 1H), 8.51, 8.36 (dt, *J* = 7.2, 3.6 Hz, 1H), 8.30, 8.16 (s, 1H), 8.30 – 8.27 (m, 1H), 7.90 (d, *J* = 3.9 Hz, 1H), 7.58 (d, *J* = 7.8 Hz, 1H), 7.34 (dd, *J* = 8.1, 2.6 Hz, 1H), 7.21 – 7.14, 7.10 – 7.04 (m, 3H), 6.98 (m, 1H), 3.07 (s, 2H), 3.03 (t, *J* = 7.6 Hz, 1H), 2.58 (t, *J* = 7.6 Hz, 1H). ^13^C NMR (125 MHz, DMSO-*d*_6_) δ 173.5, 171.4, 167.8, 149.3, 144.0, 143.9, 142.5, 139.6, 139.5, 136.3, 136.3, 130.1, 129.7, 129.5, 127.1, 127.1, 122.3, 122.2, 121.0, 118.4, 118.3, 118.2, 116.9, 116.8, 116.7, 116.5, 114.1, 113.7, 111.4, 111.4, 110.6, 110.5, 35.2, 32.9, 20.9, 20.2. HRMS calculated for [M + H]^+^ = 332.15059, found = 332.15027. HPLC purity of 96.3%, RT = 2.709 min (λ = 254 nm).

#### (E)-3-(1H-Indol-3-yl)-*N*′-(Pyridin-4-ylmethylene)­propanehydrazide (**13**)

The title compound was obtained as a light yellow solid in 69% yield (MP: 228–230 °C). ^1^H NMR (500 MHz, DMSO-*d*_6_) δ 11.66, 11.56 (s, 1H), 10.78 (s, 1H), 8.62 (d, *J* = 5.5 Hz, 1H), 8.58 (d, *J* = 5.5 Hz, 1H), 8.14, 7.95 (s, 1H), 7.61 (d, *J* = 5.5 Hz, 1H), 7.57 – 7.53 (m, 2H), 7.36 – 7.31 (m, 1H), 7.17 – 7.12 (m, 1H), 7.06 (dd, *J* = 7.6, 7.4 Hz, 1H), 6.98 (dd, *J* = 7.6, 7.2 Hz, 1H), 3.04 – 3.00 (m, 3H), 2.62 (t, *J* = 7.6 Hz, 1H). ^13^C NMR (125 MHz, DMSO-*d*_6_) δ 174.6, 168.9, 150.2, 143.4, 141.6, 141.5, 140.1, 136.3, 127.1, 127.0, 122.4, 122.3, 121.0, 121.0, 120.7, 118.4, 118.3, 118.3, 113.7, 113.5, 111.4, 35.2, 32.9, 20.6, 20.1. HRMS calculated for [M + H]^+^ = 293.13969, found = 293.13947. HPLC purity of 99.5%, RT = 4.581 min (λ = 254 nm).

#### (E)-*N*′-((1H-Indazol-5-yl)­methylene)-3-(1H-Indol-3-yl)­propanehydrazide (**14**)

The title compound was obtained as a white solid in 75% yield (MP: 245–247 °C). ^1^H NMR (500 MHz, DMSO-*d*_6_) δ 13.24, 13.21 (s, 1H), 11.30, 11.18 (s, 1H), 10.77 (s, 1H), 8.24, 8.09 (s, 1H), 8.13, 8.11 (s, 1H), 8.09 (s, 1H), 7.97, 7.92 (s, 1H), 7.81 (dd, *J* = 8.8, 1.3 Hz, 1H), 7.75 (dd, *J* = 8.8, 1.3 Hz, 1H), 7.60 – 7.53 (m, 2H), 7.33 (d, *J* = 7.8 Hz, 1H), 7.17, 7.13 (d, *J* = 2.1 Hz, 1H), 7.07 (dd, *J* = 7.5, 7.4 Hz, 1H), 6.99 (dd, *J* = 7.5, 7.4 Hz, 1H), 3.06 – 2.98 (m, 3H), 2.61 – 2.55 (m, 1H). ^13^C NMR (125 MHz, DMSO-*d*_6_) δ 173.8, 168.2, 166.7, 166.1, 146.6, 143.4, 141.9, 136.3, 136.2, 134.4, 134.3, 127.1, 127.1, 127.1, 127.0, 123.4, 122.9, 122.3, 122.2, 121.6, 121.6, 120.9, 120.9, 118.3, 118.3, 118.2, 113.9, 113.6, 111.4, 110.8, 110.8, 40.0, 39.9, 39.7, 39.5, 39.4, 39.2, 39.0, 35.2, 33.0, 20.8, 20.1. HRMS calculated for [M-H]^−^ = 330.13604, found = 330.13605. HPLC purity of 99.2%, RT = 10.170 min (λ = 254 nm).

#### (E)-*N*′-((7H-Pyrrolo­[2,3-*d*]­pyrimidin-5-yl)­methylene)-3-(1H-Indol-3-yl)­propanehydrazide (**15**)

The title compound was obtained as a white solid in 61% yield (MP: 233–236 °C). ^1^H NMR (500 MHz, DMSO-*d*_6_) δ 12.50, 12.47 (s, 1H), 11.33, 11.14 (s, 1H), 10.76 (s, 1H), 9.46, 9.31 (s, 1H), 8.84 (s, 1H), 8.30, 8.15 (s, 1H), 7.97, 7.94 (s, 1H), 7.55 (dd, *J* = 7.5, 5.0 Hz, 1H), 7.33 (dd, *J* = 8.0, 2.3 Hz, 1H), 7.17 – 7.10 (m, 1H), 7.08 – 7.03 (m, 1H), 7.00 – 6.92 (m, 1H), 3.06 (s, 2H), 3.02, 2.59 (t, *J* = 7.6 Hz, 2H). ^13^C NMR (125 MHz, DMSO-*d*_6_) δ 174.0, 168.6, 152.6, 152.3, 150.7, 150.2, 141.7, 138.8, 136.5, 136.5, 131.0, 130.8, 127.3, 127.2, 122.5, 122.4, 121.3, 121.3, 118.6, 118.6, 118.5, 118.5, 114.2, 113.8, 111.7, 111.3, 111.1, 35.3, 33.2, 21.0, 20.3. HRMS calculated for [M+Na^+^]^+^ = 355.12778, found = 355.13794. HPLC purity of 97.0%, RT = 3.403 min (λ = 254 nm).

#### (E)-*N*′-((1H-Pyrazol-4-yl)­methylene)-3-(1H-Indol-3-yl)­propanehydrazide (**16)**

The title compound was obtained as a white solid in 72% yield (MP: 210–213 °C). ^1^H NMR (500 MHz, DMSO-*d*_6_) δ 11.11, 10.98 (s, 1H), 10.75 (s, 1H), 8.08, 7.94 (s, 1H), 7.92 (s, 1H), 7.89 (s, 1H), 7.55 (t, *J* = 8.0 Hz, 1H), 7.33 (d, *J* = 8.0 Hz, 1H), 7.13, 7.10 (d, *J* = 1.8 Hz, 1H), 7.06 (t, *J* = 7.5 Hz, 1H), 6.97 (dd, *J* = 7.5, 7.4 Hz, 1H), 3.00 – 2.89 (m, 3H), 2.53 (t, *J* = 7.8 Hz, 1H). ^13^C NMR (125 MHz, DMSO-*d*_6_) δ 173.7, 168.1, 140.1, 136.9, 136.4, 136.4, 127.2, 127.1, 122.4, 121.1, 121.1, 118.5, 118.4, 118.4, 118.3, 117.1, 117.1, 114.0, 113.7, 111.5, 35.2, 33.0, 20.9, 20.2. HRMS calculated for [M+Cl^–^]^−^ = 316.09796, found = 316.09680. HPLC purity of 96.1%, RT = 2.744 min (λ = 254 nm).

#### (E)-*N*′-((1H-Pyrrolo­[2,3-*b*]­pyridin-3-yl)­methylene)-2-(1H-Indol-3-yl)­acetohydrazide (**17**)

The title compound was obtained as a white solid in 65% yield (MP: 266–268 °C). ^1^H NMR (500 MHz, DMSO-*d*_6_) δ 11.98 (s, 1H), 11.38, 11.00 (s, 1H), 10.86, 10.80 (s, 1H), 8.51, 8.44 (d, *J* = 7.8 Hz, 1H), 8.34, 8.26 (s, 1H), 8.26, 8.18 (s, 1H), 7.84 (d, *J* = 7.1 Hz, 1H), 7.60 (t, *J* = 8.5 Hz, 1H), 7.36, 7.33 (d, *J* = 8.5 Hz, 1H), 7.26, 7.20 (s, 1H), 7.17 – 7.10 (m, 1H), 7.06 (m, 1H), 6.98, 6.94 (dd, *J* = 7.8, 7.1 Hz, 1H), 4.09 (s, 1H), 3.63 (s, 1H). ^13^C NMR (125 MHz, DMSO-*d*_6_) δ 173.0, 167.6, 149.5, 149.5, 144.5, 143.7, 140.5, 136.6, 136.5, 130.8, 130.7, 130.6, 130.3, 127.8, 127.6, 124.4, 124.1, 121.6, 121.6, 119.2, 119.1, 119.0, 119.0, 117.4, 117.1, 117.0, 111.9, 110.9, 110.9, 108.9, 108.8, 32.1, 29.6. HRMS calculated for [M-H]^−^ = 316.12039, found = 316.12021. HPLC purity of 95.2%, RT = 4.210 min (λ = 254 nm).

#### (E)-*N*′-((1H-Indazol-5-yl)­methylene)-2-(1H-Indol-3-yl)­acetohydrazide (**18**)

The title compound was obtained as a white solid in 59% yield (MP: 266–270 °C). ^1^H NMR (500 MHz, DMSO-*d*_6_) δ 13.24 (s, 1H), 11.46, 11.20 (s, 1H), 10.92, 10.87 (s, 1H), 8.32, 8.11 (s, 1H), 8.13 (s, 1H), 7.97 (s, 1H), 7.88, 7.80 (d, *J* = 8.8 Hz, 1H), 7.60, 7.57 (d, *J* = 8.5 Hz, 2H), 7.38 – 7.32 (m, 1H), 7.25 (d, *J* = 6.2 Hz, 1H), 7.10 – 7.04 (m, 1H), 7.01 – 7.94 (m, 1H), 4.08 (s, 1H), 3.64 (s, 1H). ^13^C NMR (125 MHz, DMSO-*d*_6_) δ 172.5, 167.0, 147.0, 143.4, 140.5, 140.4, 136.1, 136.0, 134.4, 127.5, 127.2, 127.1, 123.9, 123.7, 123.6, 122.9, 122.9, 121.6, 121.2, 121.0, 120.9, 118.8, 118.7, 118.4, 118.3, 111.4, 111.3, 110.9, 110.8, 108.3, 31.7, 29.1. HRMS calculated for [M + H]^+^ = 318.13494, found = 318.13477. HPLC purity of 99.8%, RT = 3.624 min (λ = 254 nm).

#### (E)-*N*′-((7H-Pyrrolo­[2,3-*d*]­pyrimidin-5-yl)­methylene)-2-(1H-Indol-3-yl)­acetohydrazide (**19**)

The title compound was obtained as a white solid in 60% yield (MP: 283–285 °C). ^1^H NMR (500 MHz, DMSO-*d*_6_) δ 12.48, 12.46 (s, 1H), 11.46, 11.20 (s, 1H), 10.90, 10.84 (s, 1H), 9.44, 9.40 (s, 1H), 8.82 (d, *J* = 9.0 Hz, 1H), 8.38, 8.19 (s, 1H), 7.99 (dd, *J* = 7.2, 2.0 Hz, 1H), 7.61, 7.57 (d, *J* = 7.9 Hz, 1H), 7.34 (dd, *J* = 9.0, 8.6 Hz, 1H), 7.26, 7.21 (d, *J* = 1.5 Hz, 1H), 7.09 – 7.03 (m, 1H), 6.99, 6.93 (dd, *J* = 7.5, 7.2 Hz, 1H), 4.12, 3.64 (s, 2H). ^13^C NMR (DMSO) δ 172.5, 167.1, 152.5, 152.5, 152.4, 150.8, 150.3, 141.9, 138.6, 136.3, 136.2, 130.8, 130.7, 127.6, 127.3, 124.1, 123.9, 121.2, 121.1, 118.9, 118.8, 118.6, 118.6, 115.2, 111.5, 111.1, 111.0, 108.4, 31.8, 29.2. HRMS calculated for [M + H]^+^ = 319.13019, found = 319.13034. HPLC purity of 95.9%, RT = 4.973 min (λ = 254 nm).

#### (E)-*N*′-((1H-Pyrazol-4-yl)­methylene)-2-(1H-Indol-3-yl)­acetohydrazide (**20**)

The title compound was obtained as a white solid in 70% yield (MP: 220–223 °C). ^1^H NMR (500 MHz, DMSO-*d*_6_) δ 11.35, 10.94 (s, 1H), 10.86, 10.81 (s, 1H), 8.14, 7.95 (s, 1H), 7.93 (s, 2H), 7.59 – 7.55 (m, 1H), 7.36 – 7.30 (m, 1H), 7.24 – 7.18 (m, 1H), 7.09 – 7.03 (m, 1H), 7.01 – 6.91 (m, 1H), 3.97, 3.58 (s, 2H). ^13^C NMR (125 MHz, DMSO-*d*_6_) δ 172.9, 167.5, 140.8, 137.1, 136.4, 136.3, 127.7, 127.4, 124.4, 124.3, 121.5, 121.3, 119.1, 119.0, 118.9, 118.7, 117.4, 117.2, 111.8, 111.7, 108.6, 31.8, 29.4. HRMS calculated for [M + H]^+^ = 268.11929, found = 268.11914. HPLC purity of 97.3%, RT = 5.100 min (λ = 254 nm).

#### (E)-*N*′-((1H-Indazol-5-yl)­methylene)-3,4,5-Trimethoxybenzohydrazide (**21**)

The title compound was obtained as a white solid in 61% yield (MP: 232–235 °C). ^1^H NMR (500 MHz, DMSO-*d*_6_) δ 13.28 (s, 1H), 11.66 (s, 1H), 8.56 (s, 1H), 8.16 (s, 1H), 8.03 (s, 1H), 7.89 (d, *J* = 8.6 Hz, 1H), 7.62 (d, *J* = 8.6 Hz, 1H), 7.25 (s, 2H), 3.87 (s, 6H), 3.73 (s, 3H). ^13^C NMR (125 MHz, DMSO-*d*_6_) δ 162.5, 152.8, 148.7, 140.6, 140.4, 134.5, 128.7, 127.1, 123.8, 123.0, 121.9, 111.0, 105.2, 60.2. HRMS calculated for [M + H]^+^ = 355.14008, found = 355.13974. HPLC purity of 99.8%, RT = 3.801 min (λ = 254 nm).

#### (E)-*N*′-((1H-Pyrrolo­[2,3-*b*]­pyridin-3-yl)­methylene)-3,4,5-Trimethoxybenzohydrazide (**22**)

The title compound was obtained as a white solid in 64% yield (MP: 254–257 °C). ^1^H NMR (500 MHz, DMSO-*d*_6_) δ 12.07 (s, 1H), 11.54 (s, 1H), 8.62 – 8.55 (m, 2H), 8.30 (d, *J* = 3.2 Hz, 1H), 7.93 (s, 1H), 7.22 (s, 3H), 3.85 (s, 6H), 3.71 (s, 3H). ^13^C NMR (125 MHz, DMSO-*d*_6_) δ 182.1, 162.7, 153.1, 153.0, 149.5, 145.0, 144.4, 140.5, 130.7, 130.6, 129.2, 117.3, 117.0, 110.9, 105.3, 60.5. HRMS calculated for [M + H]^+^ = 355.14008, found = 355.13968. HPLC purity of 98.8%, RT = 3.925 min (λ = 254 nm).

#### (E)-*N*′-((1H-Indazol-5-yl)­methylene)-2-(3,4,5-Trimethoxyphenyl)­acetohydrazide (**23**)

The title compound was obtained as a white solid in 65% yield (MP: 211–213 °C). ^1^H NMR (500 MHz, DMSO-*d*_6_) δ 13.25 (s, 1H), 11.49, 11.25 (s, 1H), 8.29, 8.09 (s, 1H), 8.13 (s, 1H), 7.99 (s, 1H), 7.89, 7.81 (d, *J* = 8.8 Hz, 1H), 7.58 (dd, *J* = 7.6, 7.5 Hz, 1H), 6.66, 6.63 (s, 2H), 3.92, 3.62 (s, 2H), 3.76 (s, 3H), 3.70, 3.60 (s, 6H). ^13^C NMR (125 MHz, DMSO-*d*_6_) δ 172.2, 152.7, 147.5, 143.8, 134.5, 131.5, 123.6, 123.0, 121.9, 121.4, 111.0, 106.8, 106.5, 60.1, 56.0, 55.8. HRMS calculated for [M + H]^+^ = 369.15573, found = 369.15558. HPLC purity of 96.3%, RT = 2.709 min (λ = 254 nm).

#### (E)-*N*′-((1H-Pyrrolo­[2,3-*b*]­pyridin-3-yl)­methylene)-2-(3,4,5-Trimethoxyphenyl)­acetohydrazide (**24**)

The title compound was obtained as a white solid in 68% yield (MP: 253–256 °C). ^1^H NMR (500 MHz, DMSO-*d*_6_) δ 12.05 (s, 1H), 11.28, 11.13 (s, 1H), 8.51, 8.47 (d, *J* = 7.8 Hz, 1H), 8.35, 8.16 (s, 1H), 8.31 – 8.27 (m, 1H), 7.92 (d, *J* = 2.4 Hz, 1H), 7.22 – 7.16 (m, 1H), 6.67, 6.65 (s, 2H), 3.96, 3.45 (s, 2H), 3.77, 3.63 (s, 3H), 3.65, 3.60 (s, 6H). ^13^C NMR (125 MHz, DMSO-*d*_6_) δ 171.5, 165.8, 152.7, 152.6, 149.4, 149.3, 144.0, 143.2, 140.0, 136.0, 131.6, 130.4, 130.3, 130.0, 129.7, 116.9, 116.6, 116.4, 110.5, 110.4, 106.7, 106.4, 60.0, 60.0, 55.8, 55.7. HRMS calculated for [M + H]^+^ = 369.15573, found = 369.15442. HPLC purity of 99.0%, RT = 3.550 min (λ = 254 nm).

### SBVS, De Novo Design Campaign and Chemical Space Analysis

The SBVS procedure using the LASSBio Chemical Library^23^ was done as previously reported.^12^ For the design of the virtual compound library used in the De Novo design campaign, a workflow was constructed in KNIME. Our internal collection of reagents and fragments was first imported and converted from SMILES strings to RDKit molecules using the “RDKit From Molecule” node. Next, only those containing carboxylic acid functional groups were selected using the “RDKit Functional Group Filter” node. The resulting molecules were then filtered to remove salts and duplicates. Subsequently, the filtered structures were combined with hydrazine to generate the corresponding virtual hydrazides using the “RDKit Two Component Reaction” node with the SMARTS pattern “[#6:1](=[#8:2])­[O:3].[#7:4][#7:5]≫[#6:1](=[#8:2])[#7:4][#7:5]”. The resulting hydrazides were then reacted with the aldehydes of interest—*isoquinoline-5-carbaldehyde* (**6**), *quinoline-4-carbaldehyde* (**7**), *4-(pyridin-4-yl)­benzaldehyde* (**8**), *isonicotinaldehyde* (**9**), *1H-pyrrolo­[2,3-b]­pyridine-5-carbaldehyde* (**10**), and *1H-pyrrolo­[2,3-b]­pyridine-3-carbaldehyde* (**11**)—again using the “RDKit Two Component Reaction” node with the SMARTS pattern “[#6:1]­(=O)­[N:2]­[N:3].[#6:4]­[C:5]=O≫[#6:1]­(=O)­[N:2]/N = [C:5]/[#6:4]”. Descriptors were calculated for the resulting hydrazones using the “RDKit Descriptor Calculation” node, and the molecules were filtered based on fraction sp^3^ (≥0.1) and number of stereogenic centers (= 0). Molecular fingerprints (ECFP4, 1024 bits) were then computed using the “RDKit Fingerprint” node. The resulting bit vectors were expanded and standardized by Z-score normalization, followed by principal component analysis (PCA) to reduce the chemical space to two dimensions. A min–max normalization (0.0–1.0) was subsequently applied. Finally, the “2*D*/3D Scatterplot” node was used to visualize the chemical space, with compounds color-coded according to their fraction sp^3^ values. The small molecules were then processed by adding hydrogen atoms to their structures using the “RDKit Add Hs” node, followed by 3D coordinate generation with “RDKit Generate Coords.” The resulting geometries were optimized using the MMFF94 force field via the “RDKit Optimize Geometry” node. The optimized structures were exported in SDF format and subsequently reoptimized in Spartan’24 using the semiempirical PM6 method. The ionization states of all molecules were manually verified prior to performing the molecular docking studies against ROCK, as previously described.^12^

### Docking and MD Simulations of 18 and 21

Docking analysis of the small molecules into ROCK1 (PDB: 6E9W)^11^ and ROCK2 (PDB: 7JNT)^15^ was carried out following the procedure previously reported.^12^ For the docking studies in ROCK2 (PDB: 8GDS),^40^ the software GOLD 2025.1.0 was employed. Hydrogen atoms were added to the protein structure, and the cocrystallized ligand was extracted and used as a reference for defining the binding site, which included all amino acid residues within 8 Å of the ligand. Docking calculations were performed in the absence of water molecules using the GoldScore scoring function and the genetic algorithm with default parameters. A pharmacophore constraint was applied, consisting of two pharmacophore points: (i) Hydrogen bond acceptor—radius: 0.7 Å, constraint weight: 10; coordinates: *x* = 34.7400, *y* = 4.8230, *z* = −70.2390. (ii) Hydrogen bond donor—radius: 1.0 Å, constraint weight: 10; coordinates: *x* = 32.8370, *y* = 5.3580, *z* = −71.2780. MD simulations were performed using the FlarePro+ software (v10.0.1, Cresset, Litlington, Cambridgeshire, UK). Prior to MD, missing loops and gaps in the protein structures were reconstructed using the loop modeling tool implemented in FlarePro+, and the *N*- and *C*-terminal residues were capped with acetyl (ACE) and *N*-methylamide (NME) groups, respectively, to avoid artificial terminal charges. The resulting protein–ligand complexes were subsequently subjected to MD simulations using FlarePro+ with the OpenMM engine. MD simulations were performed employing the Open Force Field 2.2.0 for the ligands and the AMBER force field for the proteins. The systems were solvated using the TIP3P explicit water model within a truncated octahedral box, and AM1-BCC charges were assigned to the ligands. Following energy minimization, the systems underwent 200 ps of equilibration, followed by 100 ns of production simulation using a 4 fs integration time step under standard temperature and pressure conditions. Upon completion, the simulation trajectories were analyzed for RMSD and RMSF variations, as well as protein–ligand interaction profiles throughout the trajectories.

### Biochemical Assays for Kinase Inhibition

All kinase inhibition assays were performed at Reaction Biology Corporation (Malvern, PA, USA) using the radiometric HotSpot kinase assay platform. IC_50_ values were determined by curve fitting in the 10-dose IC_50_ mode, with assays conducted in duplicate using a 3-fold serial dilution factor. Assays were performed at a fixed ATP concentration of 1 μM, which is below the reported Km­(ATP) for ROCK kinases (∼10 μM). This sub-Km ATP condition was intentionally selected so that the measured IC_50_ values more closely approximate *K*_i_ values for ATP-competitive inhibition, consistent with the Cheng–Prusoff relationship *K*_*i*_ = *IC*_50_/(1+[*ATP*]/*K_m_*). Because *K*_i_ is less dependent on the ATP concentration and is often more directly comparable to theoretical/computational predictions, we preferred using ATP concentrations below Km in the biochemical assays.

### Cell Line and Cell Culture

The tumor cell line used in this study was human triple-negative breast adenocarcinoma MDA-MB-231, which lacks estrogen receptor (ER) and progesterone receptor (PR) expression, and amplification of human epidermal growth factor receptor 2 (HER2), and has both ROCK isoform overexpression, where ROCK2 is higher than ROCK1.^5^ Cell wells cultured with Dulbecco’s Modified Eagle’s Medium – High glucose (DMEM medium) from Sigma-Aldrich, cat D1152, supplemented with 10% of Fetal bovine serum (FBS) from Gibco, cat 12657–029, in a 75 cm^2^ culture flask and maintained at 37 °C with CO_2_ 5% (Thermo Scientific, Series 8000 WJ, CO_2_ incubator). Immortalized cells were obtained from Rio de Janeiro Cell Bank (BCRJ).

### Cell Viability Assay

The 3-(4,5-dimethylthiazol-2-yl)-2,5-diphenyl-tetrazolium bromide (MTT) colorimetric assay^44,45^ was performed at 30 μM final concentration per well, with 24 h incubation time. First, were seeded in 96-well plates, MDA-MB-231 cells at 2 × 10^5^ cells/mL concentration with supplemented medium and incubated. After 24 h, the controls and compounds that were previously dissolved in DMSO were added, considering a final concentration of DMSO 1% per well. After 24 h, the plate was centrifuged at 440 × g for 10 min at 4 °C (Universal centrifuge 320R Hettich), and 110 μL were discarded to add 10 μL of MTT (5 mg/mL in PBS) to each well. Then, they were incubated for 3.5 h at CO_2_ incubator. After that, 100 μL of SDS-HCl was added to dissolve the crystals of formazan, the plate was protected from light and placed for a minimum 24 h at room temperature. Finally, the absorption was read with a Molecular Devices Spectramax M5 plate reader at 595 nm. The data from 3 independent experiments tested in triplicate were analyzed using GraphPad Prism 8.0, where data are represented as mean ± SD (95% confidence interval).

### Cell-Based Scratch Assay

To evaluate the capacity of migration of the MDA-MB-231 cells after treatments with the controls and compounds of interest, the cell-based scratch assay was performed.^46,47^ First, 6 × 10^5^ cell/mL were seeded using 24-well plates with supplement DMEM medium for 24 h to reach 90 – 100% confluence. A scratch was realized using a 200 μL tip in each well, followed by 3× washed with PBS to remove nonadherent cells. After that, the compounds in final concentration per well (0.1 μM, 10 μM, 30 μM, and 100 μM with DMSO 1%) were added to pure DMEM medium with Arabinose (AraC) at a final concentration of 10^–5^ M (antimitotic reagent to inhibit proliferation). Images at 0 and 24 h were taken with a Microscope Cam 5 (Prime Cam) camera attached to a Nikon Eclipse TS2 microscope with a 4× objective lens. The scratch area was analyzed using image J and quantified using GraphPad Prism 8.0, from 3 different experiments tested in duplicate, where data are represented as mean ± SD (95% confidence interval).

### PAMPA-GIT Assays

Stock solutions (10 mM) of each test and control compound were prepared in DMSO. An aliquot of 50 μL of each solution was diluted with 4950 μL of PBS (10 mM, pH 6.6) in a 5 mL glass vial, filtered through a 0.45 μm PVDF membrane, and reserved. Acceptor plate wells were filled with 180 μL of PBS (pH 7.4):DMSO (95:5, v/v), while donor plate wells received 5 μL of a soybean L-α-phosphatidylcholine solution (20 mg/mL in dodecane). After 5 min, 180 μL of the compound solution was added in triplicate to the donor plate wells. The donor plate was then carefully placed over the acceptor plate to form a “sandwich” system, which was incubated under gentle agitation (50 rpm) for 8 h at room temperature (≈25 °C) in a closed container containing 10 mL of PBS (pH 7.4). Following incubation, the donor plate was removed, and the contents of the acceptor plate were transferred to a UV microplate for absorbance measurement using a SpectraMax reader (Molecular Devices) at the wavelength predetermined for each compound. A blank was prepared using 180 μL of PBS (pH 7.4):DMSO (95:5, v/v). The permeability result for PAMPA-GIT classifies compounds according to the percentage of absorbed fraction (Fa%), as high permeability (70–100%), medium permeability (30–69%), or low permeability.^48,49^

### PAMPA-BBB Assays

Each test and control compound (1 mg) was dissolved in 1 mL of ethanol. In a 5 mL glass vial, 1400 μL of ethanol and 3.5 mL of PBS (pH 7.4) were combined, followed by the addition of 100 μL of the ethanolic compound solution. The resulting mixture was filtered through a 0.45 μm PVDF membrane and reserved. Acceptor plate wells were filled with 180 μL of PBS (pH 7.4):ethanol (70:30, v/v), while donor plate wells received 5 μL of a porcine brain lipid solution (20 mg/mL in dodecane). After 5 min, 180 μL of the compound solution was added in triplicate to the donor plate wells. The donor plate was then carefully placed over the acceptor plate to form a “sandwich” system, which was kept undisturbed for 2 h and 45 min at room temperature (≈25 °C) in a closed container containing 10 mL of PBS (pH 7.4). Following incubation, the donor plate was removed, and the contents of the acceptor plate were transferred to a UV microplate for absorbance measurement using a SpectraMax microplate reader (Molecular Devices) at the wavelength predetermined for each compound. A blank was prepared using 180 μL of PBS (pH 7.4):ethanol (70:30, v/v). The ranges used to predict the permeability of the controls used and the compounds evaluated are a) CNS+ (prediction of high permeability through the BBB) Pe (10–6 cm·s^–1^) ≥ 4.0; b) CNS- (prediction of low permeability through the BBB) Pe (10–6 cm·s^–1^) < 2.0; c) CNS ± (method uncertainty range) 2.0 ≤ Pe (10–6 cm·s^–1^) ≤ 4.0.^50^

## Supplementary Material



## Notes

^1^H and ^13^C NMR spectrum of compound 18 at room temperature and ^1^H spectrum of 18 at 80 °C, illustrating the temperature-dependent coalescence of duplicated signals, consistent with the presence of syn and anti rotamers at the amide bond of the NAH moiety (Figures S1–S3); quantum-mechanical calculations of the amide C–N rotational barrier (ωB97-XD/6–311+G­(d,p)) supporting the dynamic equilibrium between NAH rotamers (Figure S4); conformational differences between the DFG-in (7JNT) and DFG-out (8GDS) ROCK2 structures, highlighting the αC-helix displacement and Lys121 orientation (Figure S5); additional micrographs from wound healing assays for all tested compounds (fasudil, belumosudil, 12, 17, 18, and 21) at various concentrations, evidencing dose-dependent inhibition of MDA-MB-231 cell migration (Figures S6–S12); chromatograms for 12, 17, 18, and 21 (Figures S13–S16) (PDF)

## Abbreviations

ACE: Acetyl group (*N*-terminal capping group)
ADP: Adenosine diphosphate
ATP: Adenosine triphosphate
BBB: Blood–Brain Barrier
BCRJ: Rio de Janeiro Cell Bank (Brazilian Cell Repository)
DFG: Asp-Phe-Gly (motif of the kinase activation loop)
DMEM: Dulbecco’s Modified Eagle Medium
DMSO: Dimethyl sulfoxide
DNA: Deoxyribonucleic acid
EtOH: Ethanol
FBS: Fetal bovine serum
GI: Gastrointestinal
HCl: Hydrochloric acid
HPLC: High-Performance Liquid Chromatography
HRMS: High-Resolution Mass Spectrometry
IC_50_: Half maximal inhibitory concentration
KNIME: Konstanz Information Miner (data analytics platform)
LASSBio: Laboratório de Avaliação e Síntese de Substâncias Bioativas
LIMK: LIM kinase
MD: Molecular Dynamics
MeOH: Methanol
MMFF94: Merck Molecular Force Field 94
MTT: 3-(4,5-Dimethylthiazol-2-yl)-2,5-diphenyl-tetrazolium bromide
MYPT1: Myosin phosphatase target subunit 1
NAH: *N*-acylhydrazone
NME: *N*-Methylamide group (*C*-terminal capping group)
NMR: Nuclear Magnetic Resonance
PBS: Phosphate-Buffered Saline
PCA: Principal Component Analysis
*Pe*: Effective permeability coefficient
PH domain: Pleckstrin homology domain
PM6: Parametric Method 6 (semiempirical quantum chemistry method)
QSAR: Quantitative Structure–Activity Relationship
RhoA: Ras homologue family member A
RMSD: Root Mean Square Deviation
RMSF: Root Mean Square Fluctuation
ROCK: Rho-associated coiled-coil containing kinase
SAR: Structure–Activity Relationship
SBVS: Structure-Based Virtual Screening
SDS: Sodium dodecyl sulfate
TIP3P: Transferable Intermolecular Potential with 3 Points (water model)
UFRJ: Federal University of Rio de Janeiro
