# Supplementary material for: Discovery of N‑Acylhydrazone Derivatives as ROCK Inhibitors: A Journey from Virtual Screening and Structure-Based De Novo Design to the Identification of ROCK2 Selective Inhibitors and Beyond
Source: ACS Bio Med Chem Au. 2026 Jan 27;6(2):192–209. doi: 10.1021/acsbiomedchemau.5c00246 (PMC13087806; doi:10.1021/acsbiomedchemau.5c00246)
Supplement: Supplementary file 1 [file bg5c00246_si_001.pdf]

Supporting Information

Discovery of *N*-acylhydrazone Derivatives as ROCK Inhibitors: A Journey from Virtual Screening and Structure-Based *De Novo* Design to the Identification of ROCK2 Selective Inhibitors and Beyond.

Pedro de Sena Murteira Pinheiro,[*,1,2] *Lucas Silva Franco,*[1] *Raysa Magali PillpeMeza,*[1,2] *Bárbara da Silva Mascarenhas de Jesus,*[1,2] *Gabrielli Ayumi Ito Martins,*[1] *Wesley Leandro Gouveia,*[1,2] *Daniel Alencar Rodrigues,*[3] *Marina Amaral Alves,*[4] *Lídia Moreira Lima.*[*,1,2]

1- Laboratório de Avaliação e Síntese de Substâncias Bioativas (LASSBio), Instituto de Ciências Biomédicas, Universidade Federal do Rio de Janeiro, 21941-902, Rio de Janeiro, RJ, Brazil.

2- Programa de Pós-Graduação em Farmacologia e Química Medicinal, Instituto de Ciências Biomédicas, Universidade Federal do Rio de Janeiro, 21941-902, Rio de Janeiro, RJ, Brazil.

3- School of Pharmacy and Biomolecular Sciences (PBS), Royal College of Surgeons in Ireland, 1st Floor Ardilaun House Block B 111 St Stephen's Green, Dublin 2, Ireland.

4- Walter Mors Institute of Research on Natural Products, Universidade Federal do Rio de Janeiro, 21941-902, Rio de Janeiro, RJ, Brazil.

*To whom correspondence should be addressed. E-mail: pedro.pinheiro@icb.ufrj.br; lmlima23@gmail.com.

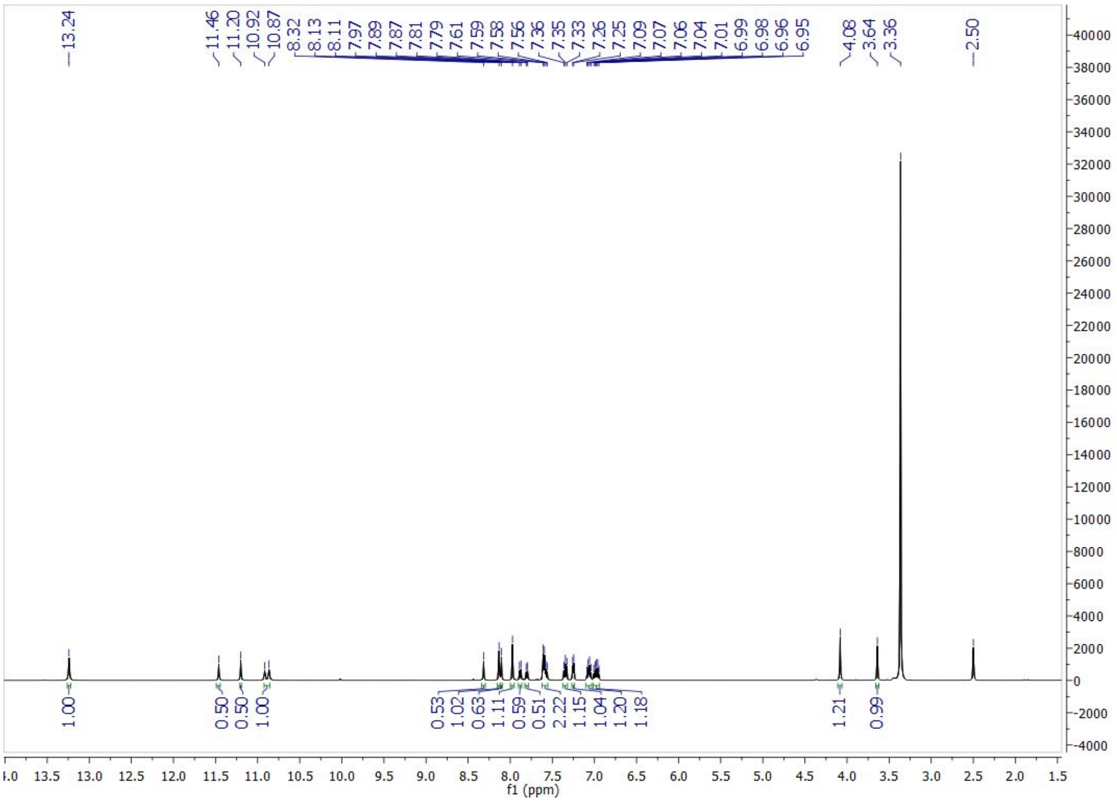

Figure S1. ^{1}H NMR spectra of **18** at room temperature in DMSO-d_6 (500 MHz).

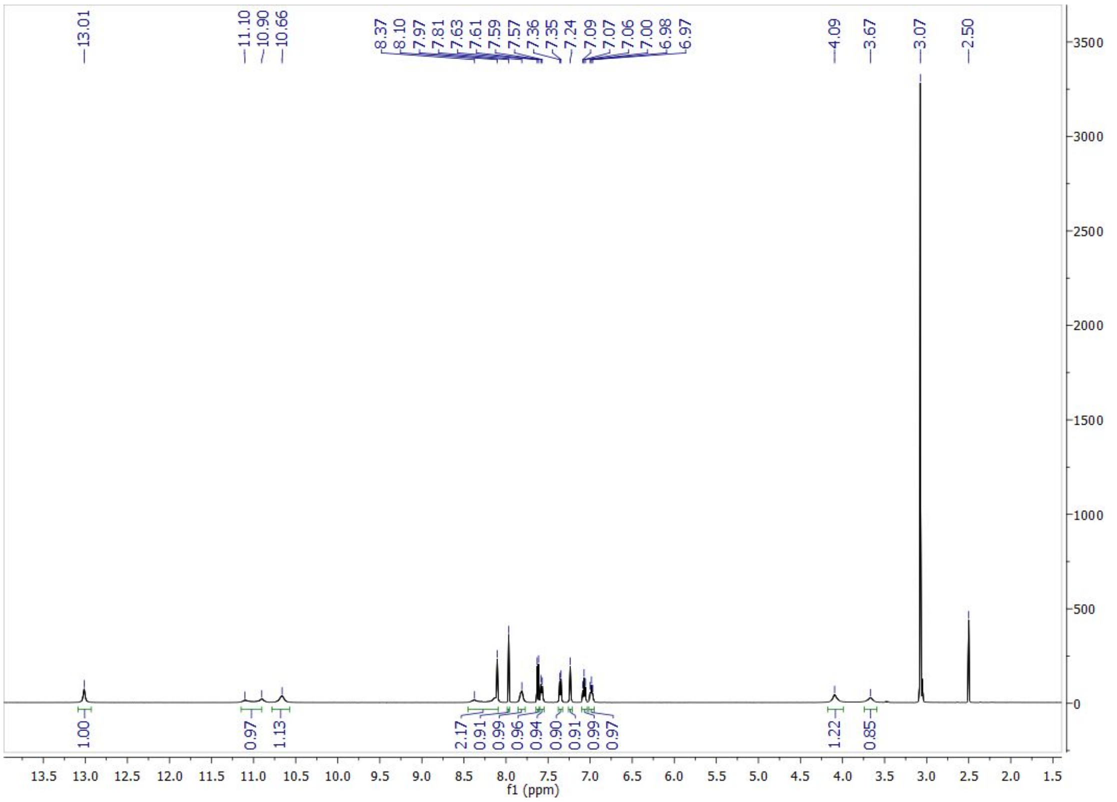

Figure S2. ^{1}H NMR spectra of **18** at 80°C in DMSO-d_6 (500 MHz).

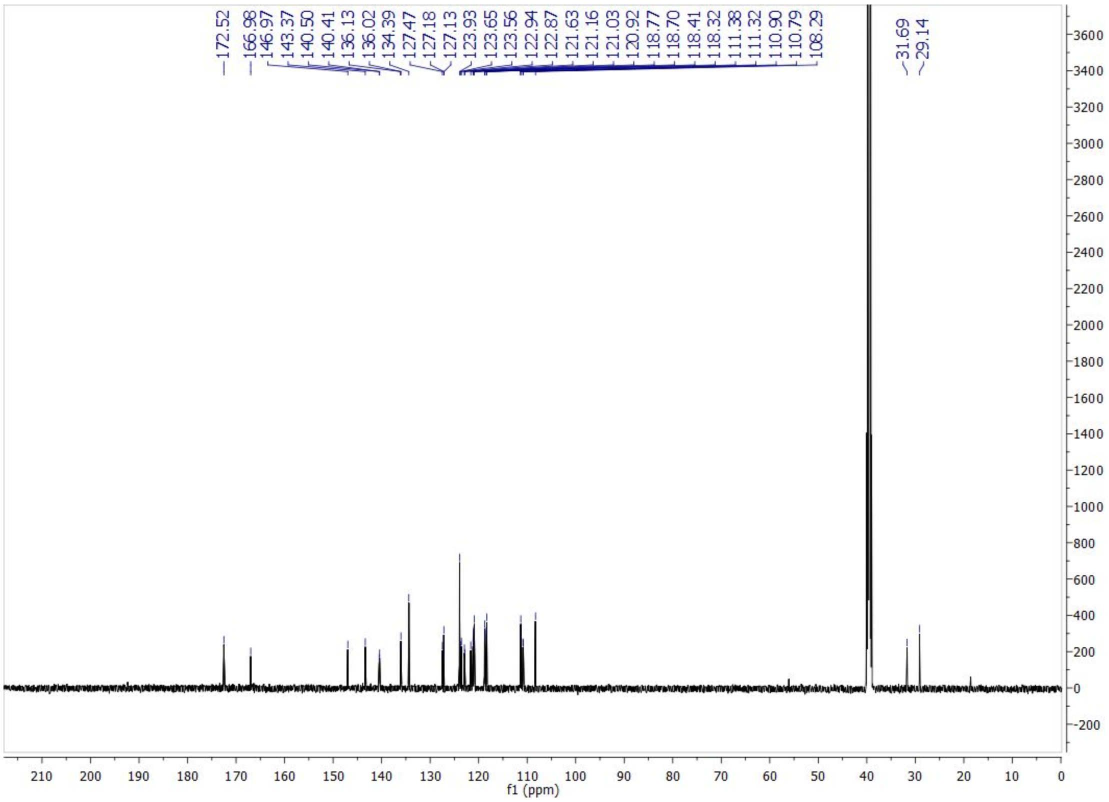

Figure S3. ^{13}C NMR spectra of **18** at room temperature in DMSO-d_6 (500 MHz).

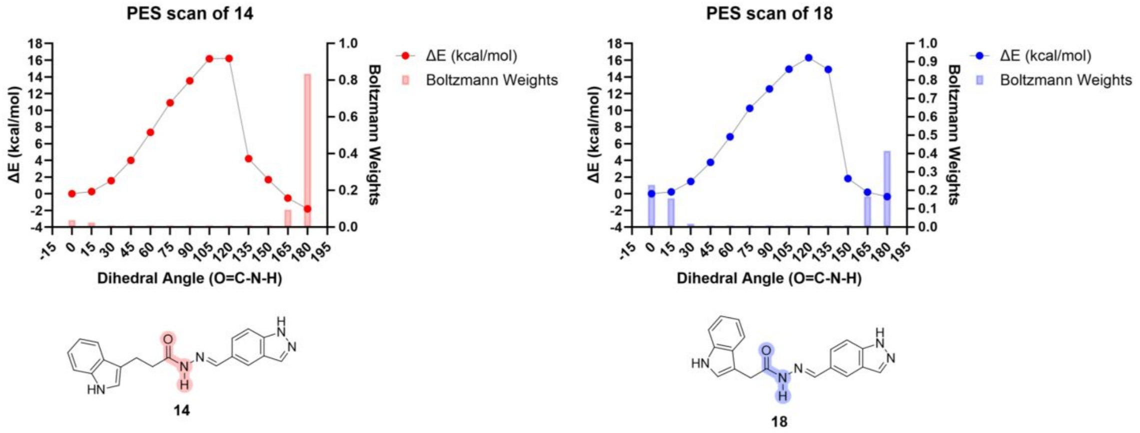

Figure S4. PES scan analysis using the ωB97-XD/6-311+G(d,p) hybrid functional for evaluation of the amide C–N bond of **14** and **18** with polar solvent effect (ε = 37.22) from 0° to 180° in steps of 15°. Calculations were done using Spartan'24.

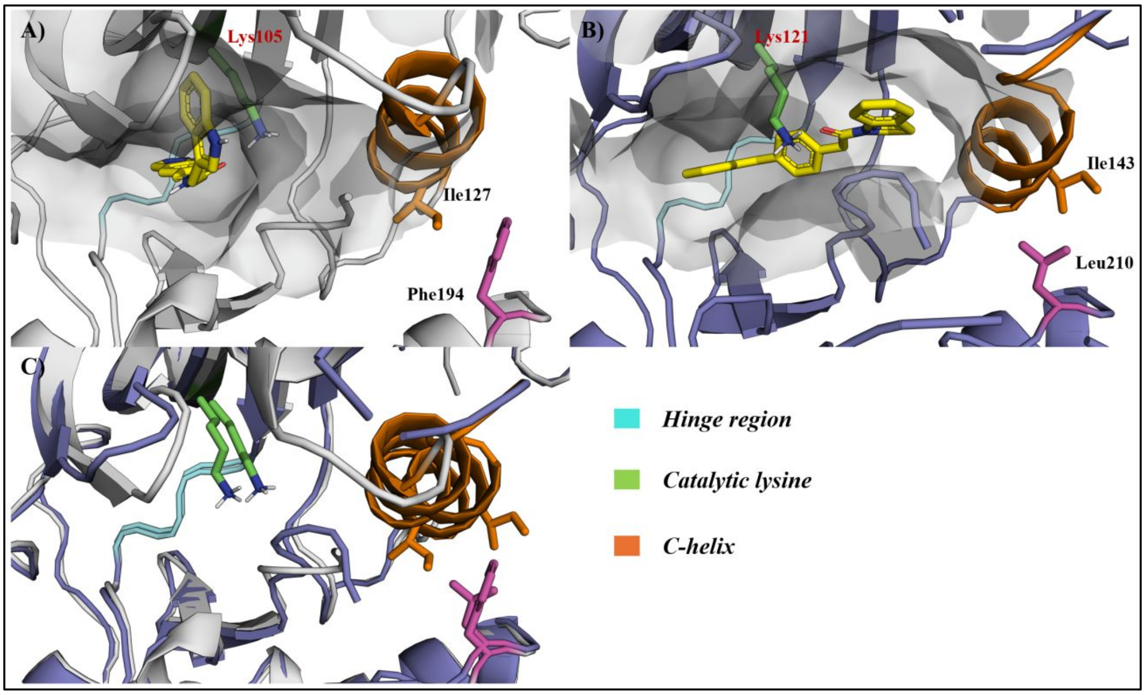

Figure S5. α*C*-helix-out conformation and accessibility to a allosteric hydrophobic pocket can occur in ROCK2 but is hindered in ROCK1 due to the residue difference L210/ ROCK2 versus F194/ROCK1. (**A**) ROCK1 α*C*-helix-in conformation (PDB: 6E9W). (**B**) ROCK2 α*C*-helix-out conformation (PDB: 8GDS). (**C**) Superposition of 6E9W and 8GDS.

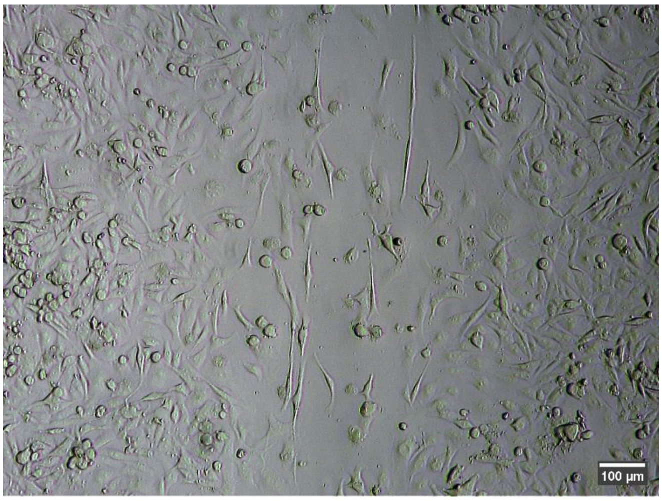

Figure S6. Micrographs of the effect on migration of the MDA-MB-231 cell line after 24 h of exposure DMSO 1%, a compound that was used as a solvent no experiment. Scale bars represent 100 µm.

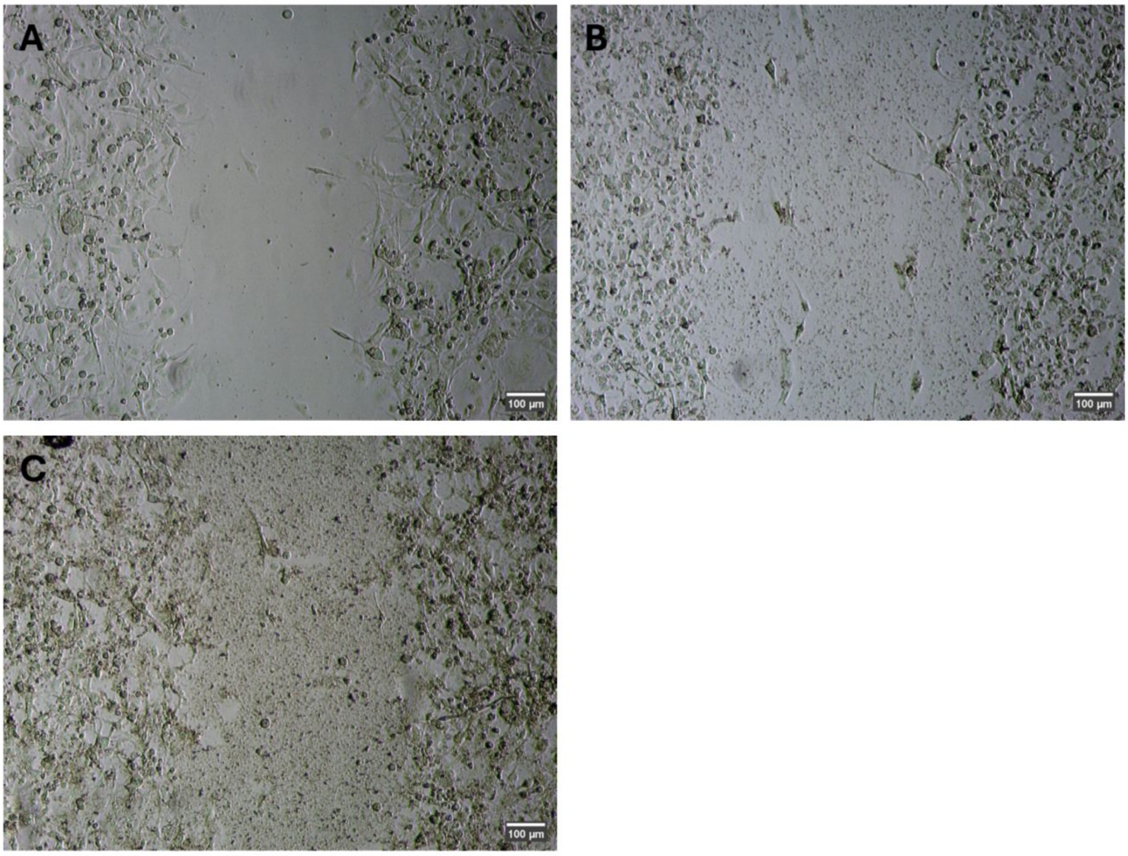

Figure S7. Micrographs of the effect on migration of the MDA-MB-231 cell line after 24 h of exposure to **12**: A) 10 μM, B) 30 μM and C) 100 μM. Scale bars represent 100 μm.

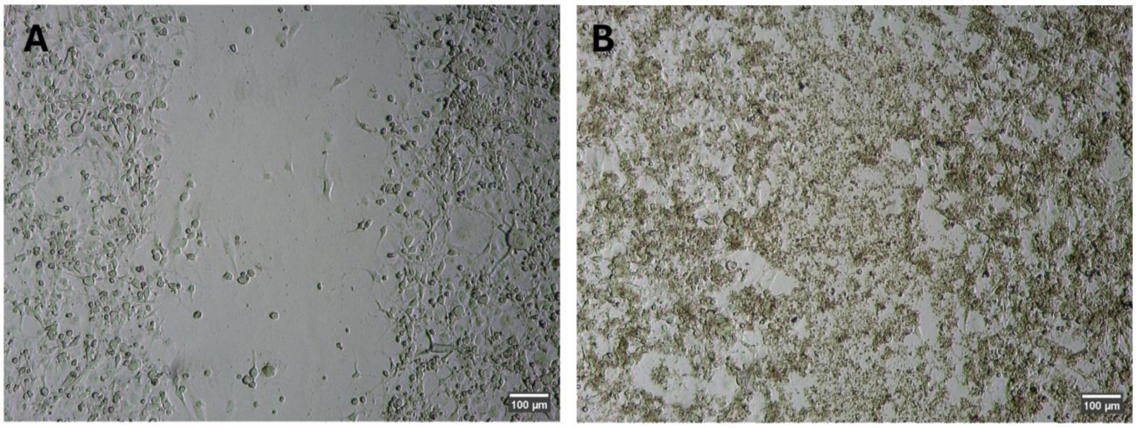

Figure S8. Micrographs of the effect on migration of the MDA-MB-231 cell line after 24 h of exposure to **17**: A) 10 μM and B) 100 μM. Scale bars represent 100 μm.

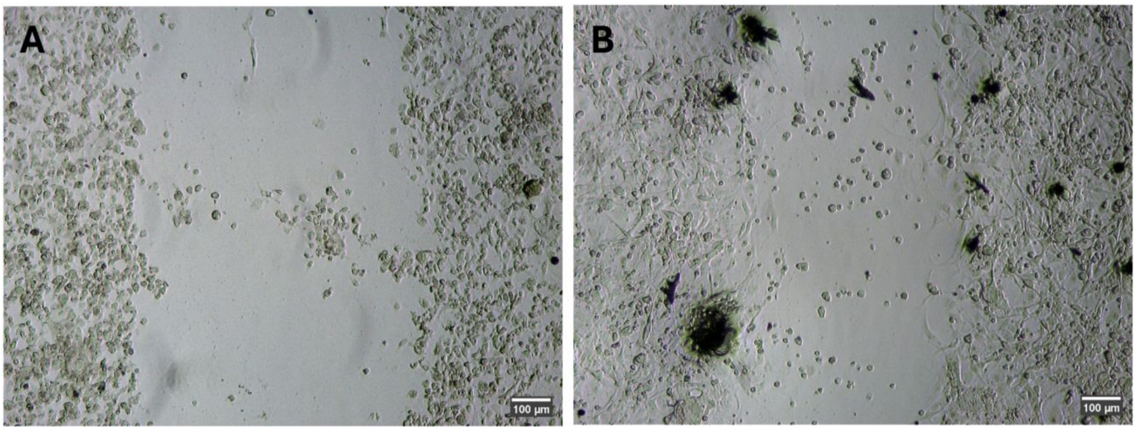

Figure S9. Micrographs of the effect on migration of the MDA-MB-231 cell line after 24 h of exposure to **18**: A) 10 µM and B) 100 µM. Scale bars represent 100 µm.

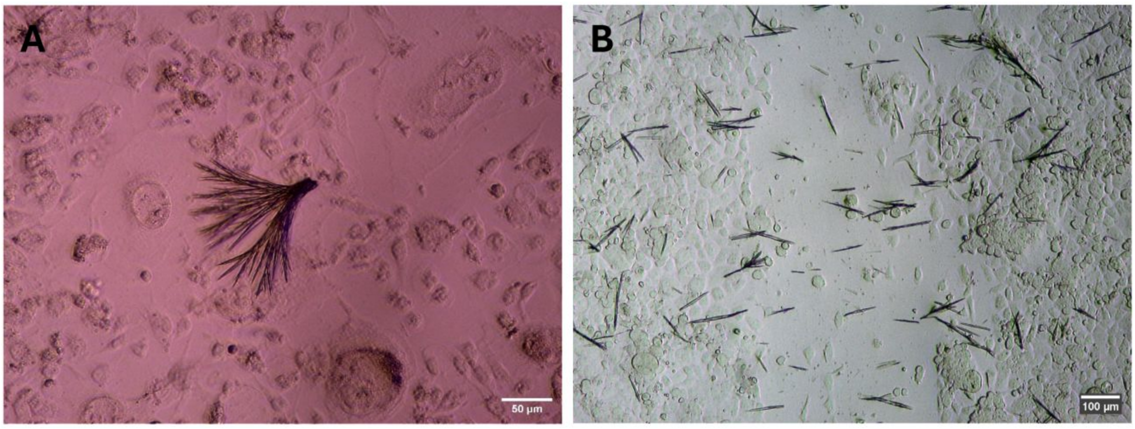

Figure S10. Micrographs of the effect on migration of the MDA-MB-231 cell line after 24 h of exposure to **21**: A) 30 µM (objective 10×), scale bar represent 50 µm and B) 100 µM (objective 4×), scale bar represent 100 µm.

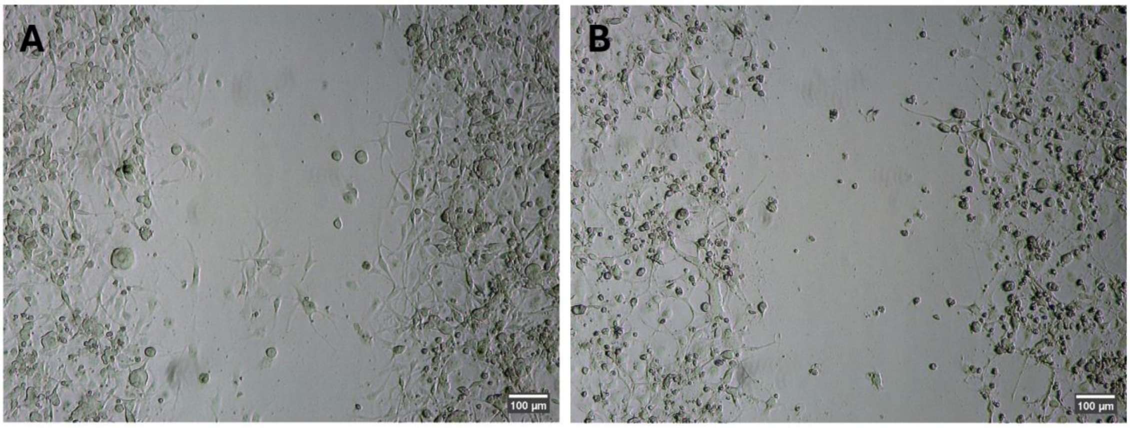

Figure S11. Micrographs of the effect on migration of the MDA-MB-231 cell line after 24 h of exposure to Fasudil: A) 30 µM and B) 100 µM. Scale bars represent 100 µm.

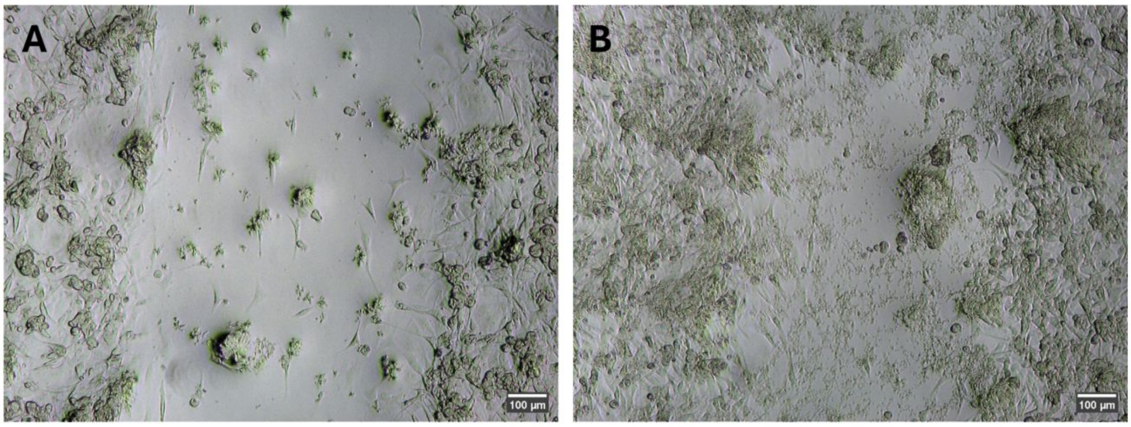

Figure S12. Micrographs of the effect on migration of the MDA-MB-231 cell line after 24 h of exposure to Belumosudil: A) 30 µM and B) 100 µM. Scale bars represent 100 µm.

SHIMADZU LabSolutions

Analysis Report

<Sample Information>

Sample Name	: INAZCOL		
Sample ID	: INAZCOL		
Data Filename	: INAZCOL.lcd		
Method Filename	: 70MEOH(B)_254nm_15min.lcm		
Batch Filename	:		
Vial #	: 1-1	Sample Type	: Unknown
Injection Volume	: 20 uL		
Date Acquired	: 11/12/2023 12:11:36	Acquired by	: System Administrator
Date Processed	: 11/12/2023 12:31:38	Processed by	: System Administrator

<Chromatogram>

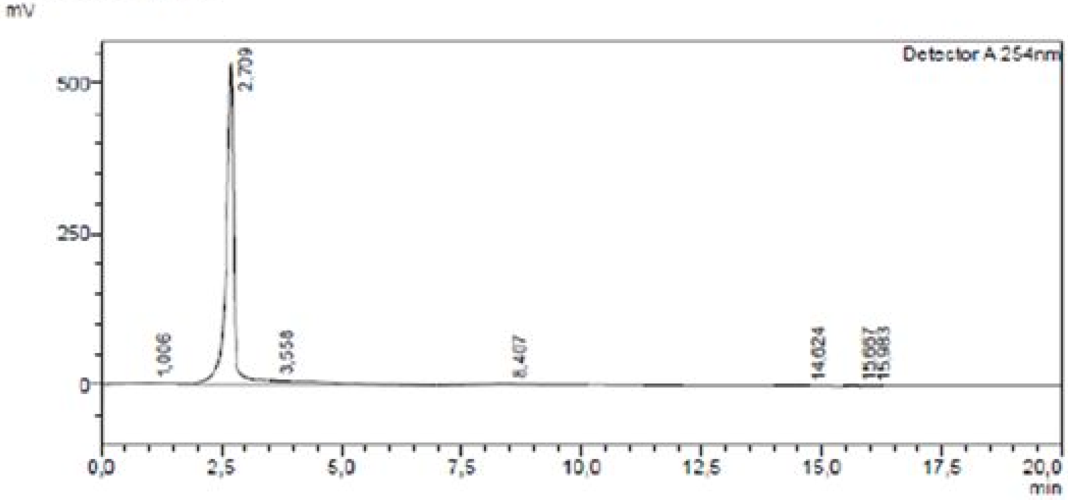

<Peak Table>

Detector A 254nm

Peak#	Ret. Time	Area	Height	Area%
1	1,006	130635	2421	1,824
2	2,709	6896559	533636	96,279
3	3,558	2093	161	0,029
4	8,407	125834	2652	1,757
5	14,624	3766	210	0,053
6	15,667	1103	102	0,015
7	15,983	3140	187	0,044
Total		7163130	539369	100,000

Detector B Ex:300nm,Em:400nm

Peak#	Ret. Time	Area	Height	Area%
Total				

Figure S13. Chromatogram of compound **12**.

SHIMADZU LabSolutions

Analysis Report

<Sample Information>

Sample Name	: indolc1aza		
Sample ID	: indolc1aza		
Data Filename	: indolc1aza.lcd		
Method Filename	: 70MEOH(B)_254nm_15min.lcm		
Batch Filename	:		
Vial #	: 1-1	Sample Type	: Unknown
Injection Volume	: 20 uL		
Date Acquired	: 25/04/2024 14:01:20	Acquired by	: System Administrator
Date Processed	: 25/04/2024 14:16:34	Processed by	: System Administrator

<Chromatogram>

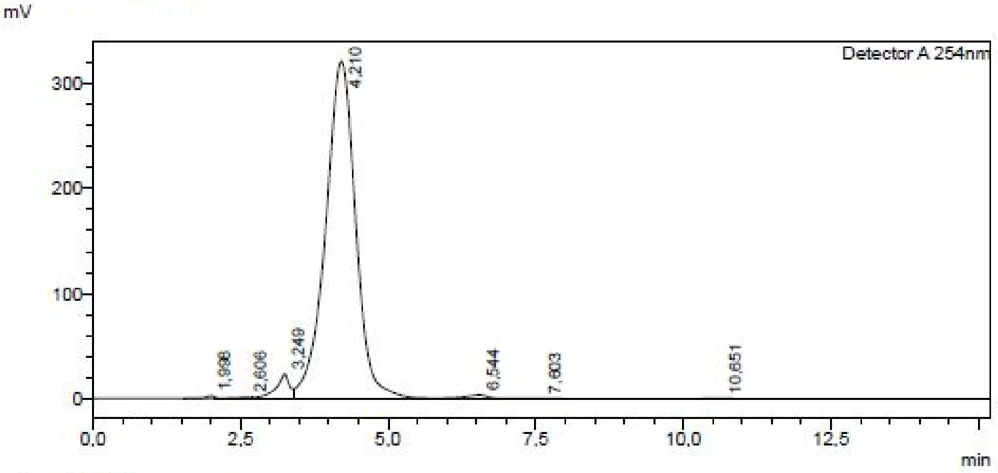

<Peak Table>

Detector A 254nm

Peak#	Ret. Time	Area%
1	1,998	0,238
2	2,606	0,198
3	3,249	3,070
4	4,210	95,250
5	6,544	0,855
6	7,603	0,238
7	10,651	0,150
Total		100,000

Detector B Ex:300nm,Em:400nm

Peak#	Ret. Time	Area%
Total		

Figure S14. Chromatogram of compound **17**.

Analysis Report

SHIMADZU LabSolutions

<Sample Information>

Sample Name	: indolc1indazol		
Sample ID	: indolc1indazol		
Data Filename	: indolc1indazol.lcd		
Method Filename	: 70MEOH(B)_254nm_15min.lcm		
Batch Filename	:		
Vial #	: 1-1	Sample Type	: Unknown
Injection Volume	: 20 uL		
Date Acquired	: 25/04/2024 13:46:54	Acquired by	: System Administrator
Date Processed	: 25/04/2024 13:59:55	Processed by	: System Administrator

<Chromatogram>

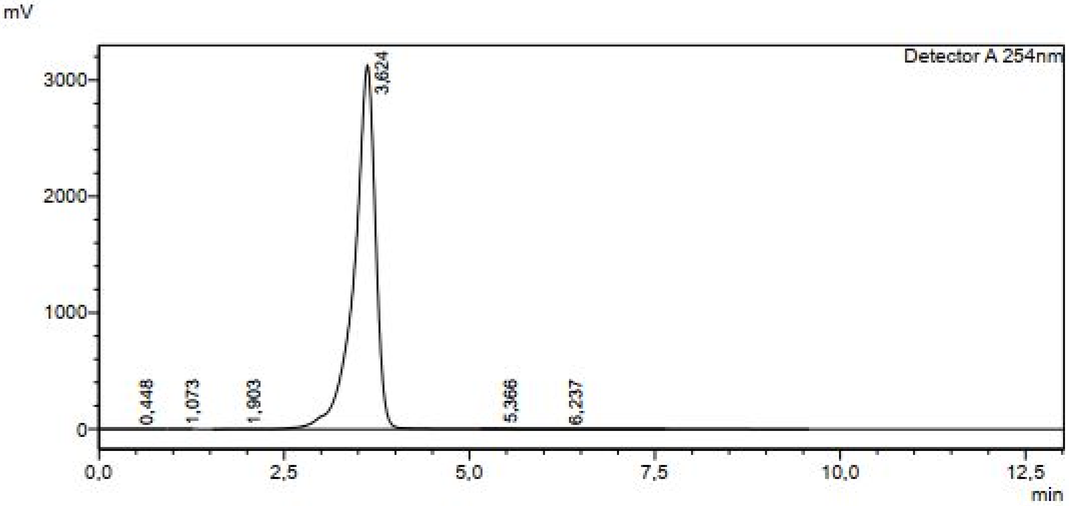

<Peak Table>

Detector A 254nm

Peak#	Ret. Time	Area%
1	0,448	0,031
2	1,073	0,004
3	1,903	0,029
4	3,624	99,888
5	5,366	0,010
6	6,237	0,038
Total		100,000

Detector B Ex:300nm,Em:400nm

Peak#	Ret. Time	Area%
Total		

Figure S15. Chromatogram of compound **18**.

SHIMADZU LabSolutions

Analysis Report

<Sample Information>

Sample Name	: TMFC0indazol		
Sample ID	: TMFC0indazol		
Data Filename	: TMFC0indazol.lcd		
Method Filename	: 70MEOH(B)_254nm_15min.lcm		
Batch Filename	:		
Vial #	: 1-1	Sample Type	: Unknown
Injection Volume	: 20 uL		
Date Acquired	: 25/04/2024 14:18:25	Acquired by	: System Administrator
Date Processed	: 25/04/2024 14:31:01	Processed by	: System Administrator

<Chromatogram>

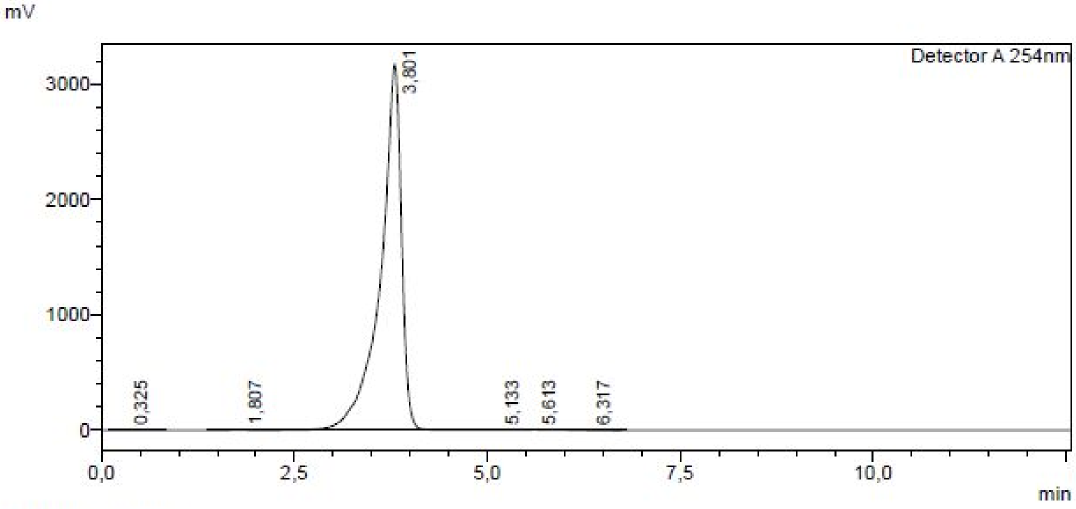

<Peak Table>

Detector A 254nm

Peak#	Ret. Time	Area%
1	0,325	0,004
2	1,807	0,026
3	3,801	99,807
4	5,133	0,008
5	5,613	0,149
6	6,317	0,007
Total		100,000

Detector B Ex:300nm,Em:400nm

Peak#	Ret. Time	Area%
Total		

Figure S16. Chromatogram of compound **21**.
